# Evolutionary dynamics under phenotypic uncertainty

**DOI:** 10.64898/2026.03.15.711953

**Published:** 2026-03-16

**Authors:** Vaibhav Mohanty, Anna Sappington, Eugene I. Shakhnovich, Bonnie Berger

**Affiliations:** 1 Department of Chemistry and Chemical Biology, Harvard University, Cambridge, MA 02138; 2 Harvard/MIT MD-PhD Program, Harvard Medical School, Boston, MA 02115 and Massachusetts Institute of Technology, Cambridge, MA 02139; 3 Program in Health Sciences and Technology, Harvard Medical School, Boston, MA 02115 and Massachusetts Institute of Technology, Cambridge, MA 02139; 4 Computer Science and Artificial Intelligence Laboratory, Massachusetts Institute of Technology, Cambridge, MA 02139; 5 Department of Mathematics, Massachusetts Institute of Technology, Cambridge, MA 02139

## Abstract

Classical population genetics has largely relied on the same stochastic differential equations (SDEs) for over 60 years to describe evolutionary dynamics. However, these SDEs ignore the fact that phenotype heterogeneity and noise are ubiquitous in biological systems from bacteria to cancers. Here, we develop Probabilistic Phenotype Genetics (ProP Gen) theory as a mathematical framework for evolutionary dynamics under phenotypic uncertainty. Our newly introduced class of SDEs show that, remarkably, phenotypic uncertainty causes many central tenets of classical population genetics to break down, such as invariance of evolutionary dynamics to global shifts in absolute fitness at fixed population size. We revisit the long-studied problem of valley crossing in rugged fitness landscapes, discovering that low-probability, high-fitness “phenotypic bridges” can substantially accelerate fitness valley crossing even at low mutation rates. We show our theory also explains a paradoxical concept we call “phenotypic buoying” whereby low-fitness phenotypes can exist at surprisingly high frequencies when carried by a high-fitness phenotype which acts as a source. ProP Gen theory uncovers complex phase diagrams of simultaneous coexistence between genotype-phenotype pairs due to phenotypic buoying, which we derive analytically exactly and verify numerically. Notably, the standard Wright-Fisher branching process is unsuitable for probabilistic phenotype numerics because it cannot correctly incorporate phenotypic uncertainty. Thus, we develop a more general, experimentally-inspired discrete-time simulation algorithm, Probabilistic Serial Dilution (ProSeD), which allows for overlapping generations, phenotypic noise, and stochastic phenotype switching. Finally, we show that the new diffusion limit of population genetics recapitulates the experimentally observed resuscitation and partitioning dynamics of bacterial “persister” strains. ProP Gen theory offers promise for describing and predicting the evolutionary dynamics of cancers, which have been empirically observed to exploit phenotypic uncertainty to evade treatment.

## 1 Introduction

Population genetics has provided robust descriptions of the stochastic fate of lineages over time, enabling both forward-time predictions of evolutionary outcomes and backward-time inference of evolutionary histories. The *diffusion limit of population genetics*, a stochastic differential equation (SDE) proposed by Kimura^1^ over 60 years ago, has had a substantial impact in computational biology and genomics, enabling evolutionary simulation methods and laying the groundwork for many central results in genetics^1–8^. The backward-time formulation of this SDE is fundamental to coalescent theory and phylogenetics^9–12^. By explicitly modeling selection-mutation balance and stochasticity via genetic drift, the diffusion limit of population genetics explains how the shape of a fitness landscape impacts the frequency trajectories of genotypes over time. In doing so, this SDE enables avenues for algorithmic prediction of fitness effects, forming the basis of many variant effect and demography estimation methods in statistical genomics^13–18^, and continues to have overarching impacts in many broad subfields of computational biology today.

Although the classical SDE provides a powerful mathematical description of how genotype frequencies change over time on a fitness landscape, it assumes that a genotype uniquely determines its phenotype and that this phenotype has a single, fixed fitness value. This assumption is at odds with empirical observations of real biological systems in which populations of a single genotype can give rise to multiple phenotypic states, often stochastically and with distinct fitness consequences^19–33^. Such phenotypic uncertainty is now recognized as a defining feature of evolution in diverse biological contexts, including bacterial persister cells^20,34–36^ and cancer cell clonal phenotypic heterogeneity^28,29,32,33,37–43^; however, little is understood as to the theoretical underpinnings of phenotypic uncertainty or its impact on evolutionary dynamics. In the setting of phenotypic uncertainty, evolution takes place not on a deterministic fitness landscape^44,45^ but rather on one in which a genotype can map probabilistically to multiple phenotypes^46–48^. Thus, the classical diffusion limit cannot represent these probabilistic mappings, nor the evolutionary consequences of phenotypic uncertainty. Accurately modeling evolution in these systems therefore requires extending population-genetic theory beyond deterministic genotype-phenotype assumptions.

Here, we develop Probabilistic Phenotype Genetics (ProP Gen) theory, a mathematical framework for evolutionary dynamics under phenotypic uncertainty of all kinds. In doing so, we introduce the first SDE that captures the nonlinear interactions of selection, mutation, and phenotypic uncertainty, which broadly encompasses phenotypic plasticity^30,32,33,39,49^, phenotypic noise^24,32,50–53^, and stochastic phenotype switching (SPS)^20,22,25,54^. Our theory predicts striking new dynamical phenomena, such as a novel dependence on absolute fitness, stabilization of low fitness phenotypes by phenotypic “buoys,” and improved fitness valley crossing via phenotypic “bridges.” To validate this theory, we introduce an evolution simulation algorithm, Probabilistic Serial Dilution (ProSeD), which can simulate probabilistic phenotype mapping, unlike the classical Wright-Fisher branching process algorithm. ProSeD simulations validate all aforementioned theoretically-predicted phenomena, including a case study in which ProP Gen theory accurately recapitulates experimentally observed bacterial persister cell resuscitation dynamics. ProP Gen theory provides a unified framework from which to study the impact of phenotypic uncertainty on evolutionary dynamics, with important implications for bacterial antibiotic resistance and cancer.

### Related Work

Although Kimura’s SDE^1^ is a mainstay of population genetics literature, mathematical modeling of evolutionary dynamics has not caught up with contemporary experimental interrogations of the role of phenotypic uncertainty in evolution. On the one hand, studies have shown overwhelming evidence that phenotypic uncertainty can drive evolution, from bacterial persister cells^20,34–36^ to cancer cell subclone phenotypic heterogeneity^28,29,32,33,37–43^. Empirically observed phenotypic uncertainty broadly manifests as phenotypic plasticity^30,32,33,39,49^, phenotypic noise^24,32,50–53,55^, and/or SPS^20,22,25,54,56^. However, few proposed models of evolution attempt to capture any form of phenotypic uncertainty. We recently introduced a *static* structural model of probabilistic genotype-phenotype (PrGP) maps^46^. Other studies have subsequently built upon our framework^47,48^, but none of these considers time-dependent evolutionary dynamics. Some simple dynamical models of SPS have added linear generators of phenotypic flux to simple exponential growth^22,34,57^, but these models cannot capture the nonlinear coupling of selection, mutation, and phenotypic uncertainty. Furthermore, to our knowledge, no model has distinguished between phenotypic noise at birth and SPS, which becomes relevant to our discussion of bacterial persister resuscitation dynamics in Section 3.4. An overview of related foundational mathematical models and the evolutionary processes they consider (e.g., selection, mutational flux, SPS, etc.) is provided in Table 1.

ProP Gen theory not only models all of these previously studied evolutionary processes, it also captures phenotypic noise at birth. Derived from a microscopic model, ProP Gen theory explicitly captures the simultaneous coupling of selection, mutation, and phenotypic uncertainty, unlike the classical diffusion limit of population genetics^1^ and current models of selection with SPS^22,34,57^. Ultimately, this unification is a crucial step in understanding real biological evolution in which mutation, selection, and many forms of phenotypic uncertainty all coincide.

## 2 Model: A new time-dependent stochastic description of evolutionary dynamics with phenotypic uncertainty

### 2.1 ProP Gen theory

**Figure 1:**
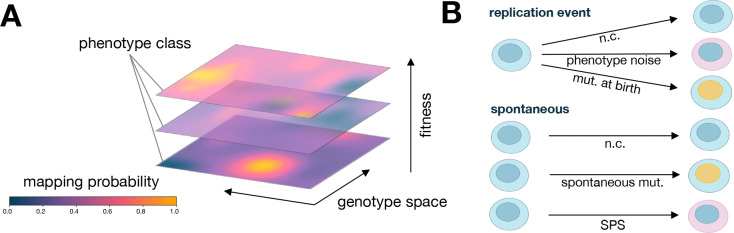
Schematic depictions of probabilistic genotype → phenotype → fitness mappings. (**A**) Diagram of a probabilistic fitness landscape. (**B**) Illustration of types of genotype mutations and phenotypic uncertainty at birth (replication event) and spontaneously. [n.c. = no change; mut. = mutation; SPS = stochastic phenotype switching.]

We now introduce the central theoretical result of this paper: a new SDE that captures selection, genetic drift, spontaneous mutations, SPS, mutations at birth, and phenotypic noise at birth simultaneously. As is standard in modern population genetics^3,61–71^, we assume a haploid asexual population of N individuals. Each individual is characterized by a genotype g∈G and phenotype p∈P, with each p associated with a Malthusian fitness (i.e., exponential growth rate), $X^{\left(p\right)}$. Traditionally, the assignment of genotype to phenotype occurs deterministically such that individuals of phenotype p will produce offspring of phenotype p exponentially according to $X^{\left(p\right)}$. In contrast, here we assume the assignment from genotype g to phenotype p is probabilistic. This converts the standard mountain-like picture of a fitness landscape^44,45^ into a “fuzzier” one; the relationship between genotype, phenotype, and fitness is better visualized as a layering of heatmaps (Figure 1A) where each genotype (horizontal coordinate) can map onto any phenotype (vertical tier) according to some probability vector (color) and replicates according to the associated fitness (height).

We next consider a dynamical process through which such haploid populations can evolve over time. At some time t, suppose there are $n_{g}^{\left(p\right)}(t)$ individuals with genotype g and phenotype p. By the definition of Malthusian fitness, after a short time δt we assume all individuals of type (g,p) have produced $\delta tX^{\left(p\right)}$ offspring. Some offspring mutate their genotype g to another genotype h with probability $\mu_{g\to h}$, while offspring from all other genotype-phenotype pairs with neighboring genotype h can mutate to genotype g with probability $\mu_{h\to g}$. We call this process *mutation at birth* or *mutation during replication*. The mutational probabilities are normalized such that outgoing probabilities sum to one: $\sum_{h\in G}\mu_{g\to h}=1$.

After offspring genotype assignment during replication, we *noisily* assign a phenotype, possibly with biased inheritance of the parental phenotype, such as in epigenetic inheritance or the partitioning of organelles in eukaryotic cell division. Phenotypic noise is captured by a probability $\varphi_{g}^{\left(k\to p\right)}$, where k is the parental phenotype and p is the offspring’s possible new phenotype. Thus, during a replication event, both the new genotype and the parental phenotype can influence the outcome of the offspring’s phenotype. We call this process *phenotype noise at birth* and regard it as a distinct type of phenotypic uncertainty. Phenotype probabilities are normalized for every genotype and parental phenotype such that outgoing probabilities of phenotype assignment sum to one: $\sum_{p\in P}\varphi_{g}^{\left(k\to p\right)}=1$.

During an organism’s lifetime, we assume heritable mutations can occur, which we call *spontaneous mutations*. The emergence of spontaneous mutations for phenotype p takes place at some rate $R^{\left(p\right)}$, with individuals chosen to mutate genotypes from g to h with probability $m_{g\to h}$. Notably, while mutation at replication and spontaneous mutation terms recombine in the deterministic SDE, they are coupled to phenotypic uncertainty in different ways. Phenotypes can also randomly switch during an organism’s lifetime due to SPS, as has been observed baker’s yeast *S. cerevisiae* ^22^ and in many bacteria such as *E. coli* ^20,36^ and *S. aureus* ^25^, which can allow them to adapt to environmental fluctuations. The SPS process takes place at some rate $S^{\left(p\right)}$, with individuals switching phenotypes from k to p according to their genotype g with a probability $\sigma_{g}^{\left(k\to p\right)}$. Because phenotypic noise at birth is coupled to both replication rate and mutation rate while SPS is not, the two sources of phenotypic changes lead to differing dynamics.

All of these processes, visualized in Figure 1B, can be combined to write $n_{g}^{\left(p\right)}(t+\delta t)$ in terms of only abundances $\left\{n_{h}^{\left(k\right)}(t)\right\}$ at time t as well as the rates and probabilities defined above. We then assume that after time δt and all of these simultaneous processes have taken place, the population re-normalizes to the original population size N, as if a bacterial culture broth were diluted according to standard laboratory serial dilution protocols. In the limit of small δt, small fitness effects, and sufficiently large N—the standard assumptions in the classical diffusion limit of population genetics—a *new* Itō SDE emerges for the dynamics of the normalized population fraction $f_{g}^{\left(p\right)}(t)$:

(2.1) $$\begin{array}{l} \frac{\partial f_{g}^{\left(p\right)}}{\partial t}=\underset{\text{selection}}{\underbrace{f_{g}^{\left(p\right)}(t)\left(X^{\left(p\right)}-\overset{‾}{X}(t)\right)}}+\underset{\text{genetic}\;\text{drift}}{\underbrace{\frac{1}{\sqrt{\tilde{N}}}\sum_{j\in P}\;\sum_{i\in G}\;\left(\delta_{gi}\delta_{pj}-f_{g}^{\left(p\right)}\right)\sqrt{f_{i}^{\left(j\right)}(t)}\eta_{i}^{\left(j\right)}(t)}}\\ \;+\underset{\text{mutation}\;\text{upon}\;\text{replication}}{\underbrace{\sum_{h\neq g}\;\sum_{k\in P}\;\varphi_{g}^{\left(k\to p\right)}X^{\left(k\right)}\left[f_{h}^{\left(k\right)}(t)\mu_{h\to g}-f_{g}^{\left(k\right)}(t)\mu_{g\to h}\right]}}+\underset{\text{spontaneous}\;\text{mutation}}{\underbrace{\sum_{h\neq g}\;\sum_{k\in P}\;R^{\left(k\right)}\left[f_{h}^{\left(k\right)}(t)\varphi_{g}^{\left(k\to p\right)}m_{h\to g}-\delta_{pk}f_{g}^{\left(k\right)}(t)m_{g\to h}\right]}}\\ \;+\underset{\text{phenotype}\;\text{noise}\;\text{at}\;\text{birth}}{\underbrace{\sum_{k\neq p}\;\left[X^{\left(k\right)}f_{g}^{\left(k\right)}(t)\varphi_{g}^{\left(k\to p\right)}-X^{\left(p\right)}f_{g}^{\left(p\right)}(t)\varphi_{g}^{\left(p\to k\right)}\right]}}+\underset{\text{stochastic}\;\text{phenotype}\;\text{switching}}{\underbrace{\sum_{k\neq p}\;\left[S^{\left(k\right)}f_{g}^{\left(k\right)}(t)\sigma_{g}^{\left(k\to p\right)}-S^{\left(p\right)}f_{g}^{\left(p\right)}(t)\sigma_{g}^{\left(p\to k\right)}\right]}}, \end{array}$$

where $\tilde{N}$ is population size scaled by generation time units, $\eta_{g}^{\left(p\right)}(t)$ represents white noise with mean $\langle \eta_{g}^{\left(p\right)}(t)\rangle =0$ and correlation $\langle \eta_{g}^{\left(p\right)}(t)\eta_{g^{\prime }}^{\left(p^{\prime }\right)}\left(t^{\prime }\right)\rangle =\delta_{gg^{\prime }}\delta_{pp^{\prime }}\delta \left(t-t^{\prime }\right),\;\delta_{gg^{\prime }}$ indicates Kronecker delta, and δ(⋅) indicates Dirac delta. We call eq. (2.1) the *Probabilistic Population Genetics (ProP Gen) equation*; it is the central result of this study and its full derivation is included in Appendix A. The ProP Gen equation is the first time a single equation has captured selection, genotype mutations, phenotypic noise, and genetic drift while also distinguishing genetic mutations and phenotype switches at birth from those occurring during an individual’s lifetime. Here, we not only subsume both Eigen’s quasispecies model^59,60^ and Kimura’s diffusion model^1,58^, but we also allow phenotypic noise during birth events to be coupled to *both* mutations and selection. To our knowledge, eq. (2.1) is the first mathematical model that explicitly captures this nonlinear coupling, which we posit is a consequence of biologically realistic and ubiquitous phenotypic uncertainty.

### 2.2 Algorithm for agent-based discrete-time evolution with phenotypic noise

We additionally introduce ProSeD (Section B, Algorithm 1), a discrete-time simulation for evolutionary dynamics under phenotypic uncertainty, to address limitations of the widely-used Wright-Fisher branching process. The classical Wright-Fisher branching process assumes a deterministic genotype-to-phenotype mapping and cannot natively incorporate phenotypic noise or SPS. In the ProSeD algorithm, when individuals reproduce their offspring undergo potential genotype mutations and then are assigned phenotypes probabilistically, incorporating phenotypic noise. The population is then downsampled to a fixed size (mimicking serial dilution) and the frequencies of all genotype-phenotype pairs are recorded, allowing for the study of their long-term evolutionary trajectories. ProSeD can also simulate SPS and phenotypic plasticity through additional probabilistic phenotype and fitness switching steps, respectively. Alongside ProP Gen theory, ProSeD allows for a more realistic and comprehensive study of evolutionary dynamics under various forms of phenotypic uncertainty.

## 3 Results

### 3.1 Low fitness phenotypes are stabilized by phenotypic buoys

#### Phenotypic buoys from a simple model of competition.

We consider the simplest possible model of competition between multiple genotypes and phenotypes: the case of two genotypes and two phenotypes, which equates to four possible genotype-phenotype pairings. Although simple, such two-state descriptions are accurate approximations in a vast array of biological systems, from binary representations of genotype mutations to gene switches to protein folding energy minima. We will show that the two-genotype, two-phenotype system yields *exact*, analytically tractable long-time population distributions that provide insight into how phenotypic uncertainty can allow less fit phenotypes to survive at high frequencies.

**Figure 2:**
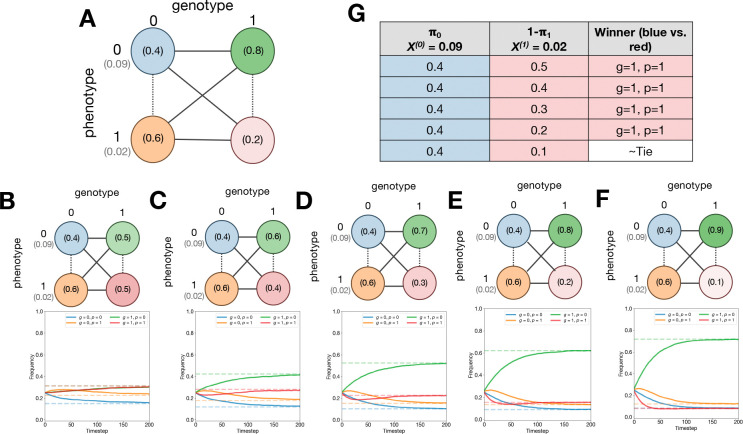
Phenotypic buoys stabilize low fitness, low probability phenotypes. (**A**) Diagram of two genotype, two phenotype model. (**B-F**) Diagrams and ProSeD simulation results for settings of 1− *π*_1_ ={0.5, 0.4, 0.3, 0.2, 0.1}. Solid lines is an averaged trajectory from 10 trials ± SE. Dashed lines are theoretically predicted equilibrium frequencies. (**G**) Table comparing equilibrium frequencies of blue (g=0,p=0) and red (g=1,p=1) nodes.

To set up the model, we assign indices of 0 and 1 to each genotype and phenotype, assuming genotypes can mutate between each other with some probability μ at each replication event and phenotype assignment only depends on the new individual’s genotype and phenotype assignment probability $\varphi_{g}^{\left(p\right)}$. Phenotype p has fitness $X^{\left(p\right)}$. This provides a setup as depicted in Figure 2A, with each genotype-phenotype pair depicted as a node in a graph.

Working in the infinite population limit (N→∞) with no spontaneous mutations or SPS, we can write down the ProP Gen equations in eq. (2.1) for this system. We then set all time derivatives to zero, since we are interested in the equilibrium population distribution. The mean fitness also becomes time-independent at equilibrium and rearranging the resulting equations, we can write down a 4 × 4 matrix eigenvalue equation for the exact equilibrium frequency vector $f^{eq}=(f_{0}^{(0),eq},f_{0}^{(1),eq},f_{1}^{(0),eq},f_{1}^{(1),eq})$. Although 4 × 4 matrix eigenvalue problems are generally difficult to solve, we prove in Appendix C that rank deficiency of the matrix allows for an exact analytical solution for the equilibrium frequencies:

(3.1) $$f_{eq}=\frac{1}{1+\frac{\alpha_{0}(2\mu -1)}{{\overset{‾}{X}}_{eq}}}\left(\begin{array}{c} \mu \pi_{0}\\ \mu \left(1-\pi_{0}\right)\\ \left[(1-\mu )+\frac{\alpha_{0}(2\mu -1)}{{\overset{‾}{X}}_{eq}}\right]\pi_{1}\\ \left[(1-\mu )+\frac{\alpha_{0}(2\mu -1)}{{\overset{‾}{X}}_{eq}}\right]\left(1-\pi_{1}\right) \end{array}\right),\;\text{with}\;{\overset{‾}{X}}_{eq}=\frac{\beta +\sqrt{\beta^{2}-4\alpha_{0}\alpha_{1}(1-2\mu )}}{2},$$

where we introduce the shorthand notation $\pi_{g}\equiv \varphi_{g}^{\left(0\right)}$ denoting the probability genotype g maps onto the higher fitness phenotype $0,1-\pi_{g}\equiv \varphi_{g}^{\left(1\right)}$ denoting the probability genotype g maps onto the lower fitness genotype, $\alpha_{g}\equiv \pi_{g}X^{\left(0\right)}+\left(1-\pi_{g}\right)X^{\left(1\right)}$, and $\beta =(1-\mu )\left(\alpha_{0}+\alpha_{1}\right)$. Simplifications of the above vector for special case parameters are discussed in Appendix C.

We then conducted numerical simulations to probe the accuracy and implications of ProP Gen theory. Consider the following thought experiment: can we, for a fixed $\pi_{0}=0.4$, intuitively predict whether genotype 1 with phenotype 1 will persist at equilibrium with a frequency higher or lower than genotype 0 with phenotype 0, given some setting of $\pi_{1}$? In Figure 2A, this is equivalent to asking whether the blue node, with mapping probability $\pi_{0}$ and fitness $X^{\left(0\right)}$, will have a higher equilibrium frequency than the red node, with mapping probability $1-\pi_{1}$ and fitness $X^{\left(1\right)}$.

If the red node has *both* lower mapping probability and lower fitness, naively we might expect that the blue node will exist at equilibrium with higher frequency. Yet, numerical simulations in Figure 2B–F show precisely the opposite result. In these simulations, we ran ProSeD (Algorithm 1) with fixed $X^{\left(0\right)},\;X^{\left(1\right)}$, and $\pi_{0}$ while sweeping over $\pi_{1}$. The population was initialized uniformly at random across the genotype-phenotype pairs and simulated until the frequencies reached equilibrium. In each trial, populations equilibrated within 250 dilutions and equilibrium frequencies matched the theoretical predictions from eq. (3.1). Over the entire $\pi_{1}$ probability sweep, summarized in Figure 2G, we found that the red node had higher equilibrium frequency than the blue node in cases where the red node’s mapping probability, $1-\pi_{1}$, was greater than blue’s, when it was equal to blue’s, *and even* when it was lower than blue’s. Only when $1-\pi_{1}$ was reduced to 0.1 did the blue and red equilibrium frequencies approach similar values.

This surprising result is explained by noting that the green node in Figure 2A–F always has high mapping probability $\pi_{1}$ and high fitness $X^{\left(0\right)}$. Thus, the green node will help absorb total population density from the remaining three nodes (discussed in mathematical depth in Appendix C.3). As it absorbs more population density, it also replicates faster, but during replication phenotype assignment is noisy. As a result, the green node acts as a sink for the blue and orange node’s population density while simultaneously acting as a source for the red node’s population density. At equilibrium, the green node’s frequency ranks highest for $\pi_{1}>0.5$ and we say that it acts as a *phenotypic buoy* for the red node, which is supported at high equilibrium frequencies despite its mapping probability and fitness being lower than the blue node’s.

This phenotypic buoying phenomenon clearly illustrates why mapping probabilities alone are not trivially predictive of equilibrium frequencies. Mapping probabilities, even when phenotypes are fully non-heritable, are ultimately *conditional* probabilities which represent the mapping probability of a phenotype given its genotype. Mapping probabilities of different phenotypes conditioned on different genotypes require priors on genotype abundances to be predictive.

#### Complex phases of coexistence for genotype-phenotype pairs.

We thus see that phenotypic buoys, even in the simplest possible model of competition between two genotypes and two phenotypes, lead to surprising equilibrium frequency distributions. Strikingly, in the presence of genotype mutations, all four genotype-phenotype pairs can persist at equilibrium due to mutations or phenotypic noise. The interplay of these two factors, which are both coupled to replication events, makes predicting the complete ordering of all four genotype-phenotype pairs at long-times a complex problem with 4! = 24 possible permutations.

We next show that the phase diagrams of coexistence between all four genotype-phenotype pairs in any arbitrary parameter regime are exactly tractable, compute the solution analytically, and provide numerical evidence through combinatorially exhaustive parameter sweeps using ProSeD (Algorithm 1). The solution follows simply from the equilibrium frequency vector solved in eq. (3.1). Frequency ordering between genotype-phenotype pairs switches at a boundary, which can be calculated by equating any two of the vector elements of $f^{eq}$. There are 42=6 possible equations, which are explicitly written and discussed in Appendix C.

**Figure 3:**
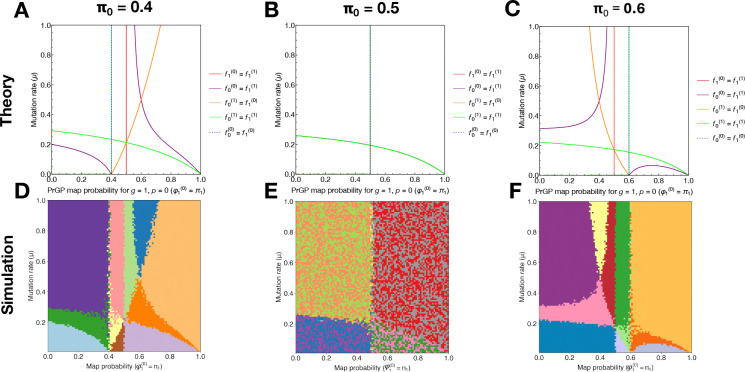
Complex phase diagrams of coexistence between genotype-phenotype pairs at equilibrium. (**A-C**) Theoretical phase boundaries for genotype-phenotype pair orderings at equilibrium for $\pi_{0}=\{0.4,0.5,0.6\}$, from the Prop Gen equation. (**D-F**) Corresponding numerical phase diagrams from ProSeD for $\pi_{0}=\{0.4,0.5,0.6\}$.

In Figure 3A–C, we plot the theoretical phase boundaries determined from eq. (3.1) as a function of μ and $\pi_{1}$. Fixing one of the phase boundary conditions $\pi_{0}=0.5$, we make three plots on the ($\pi_{1},\mu$) grid, one for each of the conditions $\pi_{1}=0.4<0.5,\;\pi_{1}=0.5$, and $\pi_{1}=0.6>0.5$. In Figure 3D–F, we plot the corresponding numerical simulation results for each setting of $\pi_{0}$ where we have empirically determined the equilibrium frequency ordering and colored the space accordingly. For a fixed value of $\pi_{0}\neq 0$, the relative ordering of $f_{0}^{(0),\text{eq}}$ and $f_{0}^{(1),\text{eq}}$ becomes fixed, reducing the maximum number of genotype-phenotype pair permutations by half, resulting in 24/2 = 12 possible orderings. The theoretical results show that only 11 sectors are expected for $\pi_{0}\neq 0$, and this is confirmed by the numerical results, aside from noise at boundaries.

Altogether, this demonstrates that predicting the ordering of genotype-phenotype pairs at long times is nontrivial. The success of our theory highlights the capability of the ProP Gen equations (eq. (2.1)) to predict the impact of phenotypic noise on evolutionary outcomes. The ProP Gen equations, and phenotypic buoy theory broadly, may shed light on how deleterious mutations can be stabilized in large populations where genetic drift is less impactful but phenotypic noise may instead strongly influence genotype and phenotype abundances. Importantly, although deleterious mutants can be “buoyed” at long times due to mutations, the phase diagrams we have calculated show that standing variation can be generated even in the *absence* of mutations. Moreover, the combined effect of mutations and phenotypic noise may be predictable from our theory.

### 3.2 Phenotypic bridges accelerate fitness valley crossing

Next, we consider a classic problem in population genetics: crossing a fitness valley^3,62,72–75^. Across numerous biological systems, there are cases in which a single mutation is deleterious and thus unlikely to persist, but a second mutation can render the resulting double mutant as fit or even fitter than the wild type. Such double mutations occur in cancers^76,77^, microbes^78,79^, and even drive pandemic waves^80,81^. In the monomorphic regime where mutations tend to fix successively, valley crossing requires extremely rare “stochastic tunneling” in which both mutations simultaneously arise during a single generation. By contrast, in the polymorphic regime populations can spread out across genotypes easily, facilitating crossing. The fitness valley crossing problem has been studied in population genetics over decades^3,62,72–75,82^, but only one study has considered the effect of phenotypic noise^83^ and it does not provide any analytical results which could systematically explain the relationship between crossing time and noise. ProP Gen theory and ProSeD, on the other hand, provide a framework for investigating and interpreting valley crossing with phenotypic noise and an *exact* result, despite the complexities arising from multiple strains competing (clonal interference), selection, mutation, and phenotypic noise.

We sought to investigate whether phenotypic uncertainty could allow mutating populations to transiently access neutral or beneficial phenotypes—even ones with low probabilities—thereby accelerating fitness valley crossing. We hypothesized that these *phenotypic bridges* could create alternative routes across rugged landscapes, effectively smoothing or bypassing deep fitness valleys and increasing accessibility of evolutionary transitions that would be improbable under strictly genotype-determined fitness.

**Figure 4:**
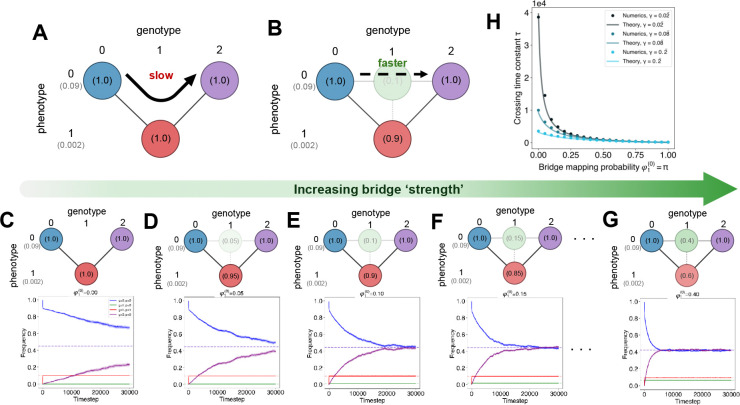
Phenotypic bridges accelerate fitness valley crossing at various depths. (**A**) Schematic of fitness valley crossing in the deterministic genotype-phenotype map setting. (**B**) Schematic of fitness valley crossing in the probabilistic genotype-phenotype map setting. (**C-G**) Schematics and time-dependent ProSeD simulation dynamics for different bridge strengths π={0.0,0.05,0.1,0.15,0.4}. Solid lines are the averages of 100 trials ± SE. (**H**) Bridge crossing/equilibration time constant τ versus mapping probability π for various valley depths γ.

Concretely, we consider a system with three genotypes (labeled 0, 1, and 2) and two phenotypes (labeled 0 and 1) with fitnesses $X^{\left(0\right)}$ and $X^{\left(1\right)}=\gamma X^{\left(0\right)}$, respectively, with γ<1. Genotypes 0 and 2 deterministically map the higher fitness phenotype 0, while genotype 1 maps with low probability $\pi \equiv \varphi_{1}^{\left(0\right)}$ to the high fitness “bridge” phenotype and with high probability $1-\pi =\varphi_{1}^{\left(1\right)}$ to the lower fitness “valley” phenotype. Mutations occur at birth, making the rate fitness-dependent, with probability of mutation μ. In the limit of π→0, the problem becomes the classic deterministic fitness valley crossing problem (Figure 4A) and equilibration across the valley is expected to be slow. For nonzero π, phenotypic noise can allow individuals to access the higher fitness phenotype and we investigate whether this increases the speed of population equilibration across the fitness valley (Figure 4B).

Using eq. (2.1) in the infinite population (N→∞) limit with no spontaneous mutations or SPS, we write the differential equations for each of the four genotype-phenotype pairs with nonzero mapping probability represented in Figure 4B as graph nodes: the starting node s, the bridge node b, the valley node v, and the ending node e:

(3.2) $$\frac{\partial f_{s}}{\partial t}\;=f_{s}\left(X_{0}-\overset{‾}{X}(t)\right)+\frac{\mu }{2}X_{0}f_{b}+\frac{\mu }{2}X_{0}\gamma f_{v}-\mu X_{0}f_{s}\;\frac{\partial f_{e}}{\partial t}\;=f_{e}\left(X_{0}-\overset{‾}{X}(t)\right)+\frac{\mu }{2}X_{0}f_{b}+\frac{\mu }{2}X_{0}\gamma f_{v}-\mu X_{0}f_{e}\;\frac{\partial f_{b}}{\partial t}\;=\pi \left(f_{b}X_{0}+f_{v}X_{0}\gamma \right)-f_{b}\overset{‾}{X}(t)+\pi \mu X_{0}\left(f_{s}+f_{e}\right)-\pi \mu \left(f_{b}X_{0}+f_{v}X_{0}\gamma \right)\;\frac{\partial f_{v}}{\partial t}\;=(1-\pi )\left(f_{b}X_{0}+f_{v}X_{0}\gamma \right)-f_{v}\overset{‾}{X}(t)+(1-\pi )\mu X_{0}\left(f_{s}+f_{e}\right)-(1-\pi )\mu \left(f_{b}X_{0}+f_{v}X_{0}\gamma \right)$$

In Appendix D, we calculate the exact equilibrium frequency vector $f^{\text{eq}}$ analytically. We then linearize the above nonlinear equations around the equilibrium vector and find the rate of the slowest exponential decay mode by determining the lowest magnitude eigenvalue of the Jacobian. The associated bridge equilibration time constant τ is then the reciprocal of the slowest rate; we are able to calculate an exact analytical result:

(3.3) $$\tau =\frac{2}{\sqrt{\left(1\;+\;\theta {)}^{2}(1\;-\;\mu {)}^{2}\;-\;4\theta (1\;-\;2\mu \right)}\;-\;(1\;-\;\theta )(1\;-\;\mu )},\;\text{with}\;\theta \equiv \pi +\gamma (1\;-\;\pi ).$$

To validate this theory, we run ProSeD for various bridge probabilities π with an initial population confined to the deterministic node at genotype 0. We then allow the population to relax toward equilibrium; the population fraction on genotype 0 decays and the population fraction on genotype 2 increases towards their respective equilibrium values. Figure 4C–G displays averaged stochastic trajectories from ProSeD simulations for various bridge probabilities π. We observe faster relaxation toward equilibrium even for very low probability bridges. For instance, even with only a 5% chance of mapping onto the high fitness bridge, exponential decay is notably quicker (Figure 4D). To quantify the relationship between the decay time constant and bridge probability, we use ordinal distance regression to estimate the exponential decay rate for the observed trajectory of $f_{e}(t)$, the increasing population fraction on genotype 2. The reciprocal of this rate is the empirical bridge crossing time constant τ, which we compare to the theory in eq. (3.3) above.

In Figure 4H, we observe excellent agreement between theory and numerical simulations for τ versus π across various fitness valley depths γ. We emphasize that the theoretical curves are fully determined by the simulation parameters and are not fit to data points, validating our ProP Gen equations (eq. (2.1)) and the derived equilibration time constant τ in eq. (3.3) as accurately describing bridge dynamics. Notably, we quantitatively observe a rapid decay of τ versus π and find that the effect of π is larger for deeper fitness valleys (smaller γ; Figure 4H).

Importantly, our theoretical predictions and numerical validation demonstrate that even *weak* phenotypic bridges can substantially accelerate fitness valley crossing in the polymorphic regime, where many mutants compete with each other and generations overlap. We are able to show this analytically with highly accurate, zero-fit theoretical curves validated by ProSeD simulations. Thus, our theoretical framework provides a robust starting point for modeling how infectious diseases or cancers may exploit phenotypic noise to bypass fitness valleys imposed by immunological or therapeutic defenses.

### 3.3 Mean fitness can decrease with time

#### Absolute, not just relative, fitness impacts population dynamics.

**Figure 5:**
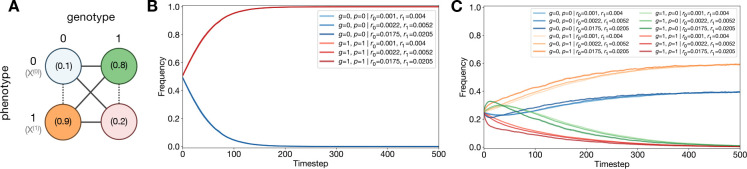
Phenotypic uncertainty leads to dependence on absolute fitness. (**A**) Schematic showing genotype-phenotype pairs and their mapping probabilities, $\phi_{g}^{\left(p\right)}$, in the setting of phenotypic uncertainty. In the deterministic setting, $\phi_{0}^{\left(0\right)}=1$ and $\phi_{1}^{\left(1\right)}=1$. (**B**) Frequencies over time for deterministic genotype-phenotype pairs at different absolute, but the same relative, fitnesses. (**C**) Frequencies over time for probabilistic genotype-phenotype pairs at different absolute, but the same relative, fitnesses. Note $r_{i}\propto X^{\left(p\right)}$ as it is the per-generation reproduction probability for a given phenotype p used in the ProSeD simulations.

Notably, uncertain genotype-phenotype mapping leads population dynamics to depend on absolute, not simply relative, fitnesses. In Appendix E.1, we show mathematically that shifting the entire fitness landscape by some absolute fitness A does not transform the selection term in the classic, deterministic SDE but that it does change the probabilistic SDE. Specifically, shifting the fitness landscape by A introduces a dependence of the probabilistic SDE selection term on A, demonstrating that absolute fitness affects population dynamics under phenotypic uncertainty. Running ProSeD evolutionary dynamics simulations at different settings of absolute fitness, while keeping relative fitness constant, demonstrates this behavior. In Figure 5A–C, we see that although shifts in absolute fitness do not affect dynamics in the deterministic setting, they do significantly alter dynamics in the presence of phenotypic uncertainty. Eigen’s quasispecies model has a similar feature, but here we note that phenotypic uncertainty can cause this dependence even in the absence of mutations.

#### Phenotypic uncertainty can transiently decrease mean fitness.

**Figure 6:**
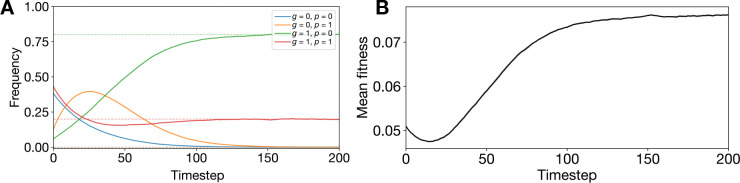
Mean fitness can decrease from phenotypic uncertainty alone. (**A**) Frequency over time of genotype-phenotype pairs with parameter settings detailed in the main text. (**B**) Mean fitness over time for entire population.

Moreover, we can construct examples in which the change in mean fitness can be negative over time, even in the setting of no genotype mutations. In other words, phenotypic uncertainty alone can cause mean fitness to decrease. For instance, if we let $X^{\left(0\right)}=0.09,\;X^{\left(1\right)}=0.02,\;\varphi_{0}^{\left(0\right)}=0.1,\;\varphi_{0}^{\left(1\right)}=0.9,\;\varphi_{1}^{\left(0\right)}=0.8$, and $\varphi_{1}^{\left(1\right)}=0.2$ with initial frequencies $f_{0}^{\left(0\right)}=0.4,\;f_{0}^{\left(1\right)}=0.1,\;f_{1}^{\left(0\right)}=0.05$, and $f_{1}^{\left(1\right)}=0.45$, we see in Figure 6A–B and Appendix E.2 that the mean fitness initially decreases. Genotype 0, phenotype 1 (orange) has relatively high mapping probability but low fitness, thus its frequency will be low asymptotically. However initially, its frequency grows due to the relatively high initial frequency of genotype 0, phenotype 0 (blue). The rapid growth of genotype 0, phenotype 1 and non-monotonic behavior contributes to the initial decrease in mean fitness before its eventual increase towards its equilibrium value (Figure 6B). Other evolutionary scenarios (such as specific mutation matrices) can cause transient decrease in mean fitness, but our focus here is to demonstrate that phenotypic uncertainty can cause this decrease even in the absence of mutations.

### 3.4 Dynamics of bacterial persister resuscitation

We next sought to apply ProP Gen theory to model the resuscitation of bacterial persister cells using experimentally determined SPS parameters^36^, finding that the ProP Gen equations recover empirically observed dynamics^36^. Persister cells are bacterial phenotypic, but not genotypic, variants which are able to survive in the face of environmental stress such as antibiotic treatment. In normal environmental conditions, these cells are less fit than the wild type phenotypic variant and thus reproduce more slowly, but SPS or phenotypic plasticity induced by environmental stress generates a nonzero proportion of these less fit variants. Persister cells enter a state of dormancy and can “resuscitate” after the stressor has been removed.

In Fang and Allison’s^36^ experimental study, dormant *E. coli* and *S. enterica* persister cells resuscitated with an exponential rate after antibiotics were washed away, resuming cell division with varying degrees of success. Damaged persisters carried morphological defects and sometimes were unable to replicate or replicated more slowly and at times “partitioned” to produce damaged offspring and healthy offspring^36^. Successfully reactivated persisters with no morphological damage continued to replicate at baseline rates, producing healthy offspring. The fine-grained time resolution showed that damaged cells were rapidly outnumbered by healthy replicating cells within hours of resuscitation, explaining why previous studies with coarser time measurements had never observed such partitioning.

Here, we show that the ProP Gen equations can be exactly solved for persister resuscitation dynamics, which highlights the distinction between SPS and phenotypic noise at birth. In the experiments, no population bottleneck is imposed, so we ignore genetic drift and remove the mean fitness $\overset{‾}{X}(t)$ term from the selection portion of eq. (2.1). We write the system of ODEs for absolute abundances $\left\{n_{i}(t)\right\}$ of dormant persisters (P), resuscitated cells which fail replication (F), resuscitated cells which are damaged but can replicate (D), and resuscitated healthy cells (H):

(3.4) $$\begin{array}{l} \frac{\partial n_{P}}{\partial t}=-S(t)n_{P}(t),\;\frac{\partial n_{D}}{\partial t}=X^{\left(D\right)}\left(1-\varphi^{\left(D\to H\right)}\right)n_{D}(t)+S(t)\sigma^{\left(P\to D\right)}n_{P}(t),\\ \frac{\partial n_{F}}{\partial t}=S(t)\sigma^{\left(P\to F\right)}n_{P}(t),\;\text{and}\frac{\partial n_{H}}{\partial t}=X^{\left(H\right)}n_{H}(t)+X^{\left(D\right)}\varphi^{\left(D\to H\right)}n_{D}(t)+S(t)\sigma^{\left(P\to H\right)}n_{P}(t), \end{array}$$

where $S(t)=\alpha e^{\beta t}$ is the exact form of the persister cell resuscitation rate experimentally supported by Fang and Allison^36^, $\sigma^{P\to i}$ for i∈{D,F,H} represents SPS from dormancy to the three resuscitated phenotypes, $X^{\left(p\right)}$ are fitnesses, and $\varphi^{D\to H}$ is the probability that the offspring of a damaged cell is healthy. We show in Appendix F that the above system of equations yields the exact analytical solution

(3.5) $$\begin{array}{l} n_{P}(t)=n_{P}(0)e^{-c\left(e^{\beta t}-1\right)},\;n_{F}(t)=n_{F}(0)+\sigma^{\left(P\to F\right)}\left[n_{P}(0)-n_{P}(t)\right],\\ n_{D}(t)=e^{at}\left\{n_{D}(0)+\alpha \sigma^{\left(P\to D\right)}n_{P}(0)\beta^{-1}e^{c}c^{-q_{D}}\left[\gamma \left(q_{D},ce^{\beta t}\right)-\gamma \left(q_{D},c\right)\right]\right\}\\ n_{H}(t)=e^{X^{\left(H\right)}t}\left\{n_{H}(0)+kn_{D}(0)+\alpha Bn_{P}(0)\beta^{-1}e^{c}c^{-q_{H}}\left[\gamma \left(q_{H},ce^{\beta t}\right)-\gamma \left(q_{H},c\right)\right]\right\}\\ \;-ke^{at}\left\{n_{D}(0)+\alpha \sigma^{\left(P\to D\right)}n_{P}(0)\beta^{-1}e^{c}c^{-q_{D}}\left[\gamma \left(q_{D},ce^{\beta t}\right)-\gamma \left(q_{D},c\right)\right]\right\}, \end{array}$$

where we have defined shorthand $a=X^{\left(D\right)}\left(1-\varphi^{\left(D\to H\right)}\right)$, $c=\frac{\alpha }{\beta }$, $q_{D}=1-\frac{a}{\beta }$, $q_{H}=1-\frac{X^{\left(H\right)}}{\beta }$, $k=\frac{X^{\left(D\right)}\varphi^{\left(D\to H\right)}}{X^{\left(H\right)}-a}$, $B=\sigma^{\left(P\to H\right)}+k\sigma^{\left(P\to D\right)}$, and γ(s,x) is the lower incomplete gamma function.

**Figure 7:**
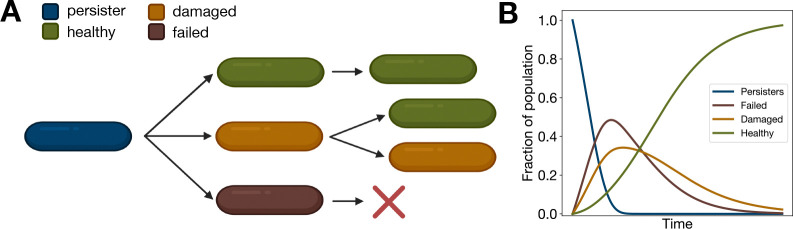
Persister cell SPS case study. (A) Diagram of empirically observed SPS transitions for dormant *E. coli* and *S. enterica* persister cells^36^. All cells have the same genotype. (B) Dynamics of bacterial resuscitation as modeled by eq. (3.5), recapitulating the experimentally observed^36^ transient appearance of damaged and failed resuscitated cells.

In Figure 7, we plot normalized time-dependent trajectories for each of the four phenotypes, provided by eq. (3.5). The dynamics from our theory clearly capture the non-monotonic abundances of failed and damaged persisters, which peak temporarily before disappearing. This transient appearance of abnormal phenotypes accurately recapitulates the experimental findings of Fang and Allison^36^ and explains why prior experiments, which tracked persister resuscitation with coarse temporal resolution, failed to capture these damaged cells. Encouragingly, ProP Gen theory is able to model and predict fine-grained dynamical phenomena observed in experiments stemming from phenotypic uncertainty, showing that the SDE possesses mechanistic fidelity.

## 4 Discussion

We introduced ProP Gen theory, a unified theoretical framework that generalizes the classical diffusion limit of population genetics to explicitly incorporate the interplay of selection, mutation, and phenotypic uncertainty. By allowing each genotype to map to a distribution over phenotypes with potentially distinct fitnesses, switching rates, and replication-coupled noise, our formulation captures a rich set of biological processes that have remained inaccessible to traditional models. Through exact analytical solutions, numerical simulations, and *E. coli* and *S. enterica* persister cell experimental case study, we find that phenotypic uncertainty fundamentally reshapes evolutionary dynamics, altering long-held assumptions about fitness valley crossing and adaptation.

Our theory uncovers two striking new dynamical phenomena caused by phenotypic uncertainty: phenotypic buoying and phenotypic bridging. Phenotypic buoys occur when a high-fitness phenotype stabilizes the persistence of markedly less fit phenotypes, even in the absence of genotype mutations. Exact analytical solutions of the two-genotype, two-phenotype model show that low-fitness phenotypes can dominate at equilibrium when coupled to a strong phenotype source. This finding shows that standing variation in phenotypes can be generated even in the absence of mutations and provides exact results for expected long-term genotype-phenotype distributions when the forces of selection, mutation, phenotypic noise balance each other. In the context of fitness valley crossing, we find that even rare expression of a high fitness phenotype creates phenotypic bridges, alternative transient routes that drastically accelerate equilibration over and crossing between fitness valleys. The exact time constants derived from our diffusion limit match our numerical simulations without parameter fitting, demonstrating the accuracy of our theory. We note the effect of phenotypic bridges is particularly pronounced in deep valleys, suggesting that phenotypic noise may enable highly unlikely evolutionary transitions, including those underlying immune escape in viruses or therapy evasion in cancers. Finally, we find that the experimentally observed transient appearance of damaged cells in bacterial persister resuscitation dynamics can be aptly described by the same ProP Gen model.

Together, these findings highlight that evolution in realistic biological systems cannot be fully understood without explicitly considering phenotypic uncertainty. Populations explore an effective fitness landscape that cannot be fully described by classical deterministic genotype-fitness maps; instead, a PrGP map coupled with ProP Gen theory captures previously unforeseen complexity. We foresee ProP Gen theory offering a promising model by which to explore the effects of phenotypic uncertainty and make testable predictions to be experimentally validated with high-throughput paired genotype-phenotype time series data, which the field currently lacks.

Overall, our work establishes a mathematically rigorous and biologically grounded extension of classical population genetics to incorporate phenotypic uncertainty. By revealing how phenotypic uncertainty reshapes equilibrium and transient adaptive behaviors, ProP Gen opens the door to more complete predictive models of evolutionary dynamics in microbial populations, cancer progression, and beyond.

## Supplementary Material

Supplement 1

## Figures and Tables

**Table 1:** Table of foundational papers introducing population genetics differential equations. This work is the first to incorporate explicit nonlinear coupling of selection, mutation, and phenotypic noise.

Paper/book	Model	Selection	Genetic drift	Mutational flux	SPS	Mutations AB*	Phenotype noise AB*
Kimura, 1962^1^	Diffusion limit with drift	✓	✓				
Crow and Kimura, 1970^58^	Diffusion limit with mutations	✓	✓	✓			
Eigen, 1971^59^; Eigen et al., 1988^60^	Quasispecies equation, infinite population	✓				✓	
Thattai and van Oudenaarden, 2004^57^; Acar et al., 2008^22^	SPS with growth	✓**			✓		
**This work**	**All of the above, plus phenotype noise AB**	✓	✓	✓	✓	✓	✓

* No assumptions on the weakness of selection/mutation.

** unbounded exponential growth, no bottleneck/serial dilution. [SPS = stochastic phenotype switching. AB = at birth.]

## Code availability.

Code for simulations and plots: https://github.com/vastcollab/ProPGen.
