# Supplementary material for: Evolutionary dynamics under phenotypic uncertainty
Source: bioRxiv. 2026 Mar 16:2026.03.15.711953. Preprint. [Version 1] doi: 10.64898/2026.03.15.711953 (PMC13015399; doi:10.64898/2026.03.15.711953)
Supplement: Supplement 1 [file NIHPP2026.03.15.711953v1-supplement-1.pdf]

bioRxiv preprint doi: https://doi.org/10.64898/2026.03.15.711953; this version posted March 16, 2026. The copyright holder for this preprint (which was not certified by peer review) is the author/funder, who has granted bioRxiv a license to display the preprint in perpetuity. It is made available under aCC-BY 4.0 International license.

Accepted to the 30th Annual International Conference on Research in Computational Molecular Biology (RECOMB), 2026

Supplementary Information for "Evolutionary dynamics under phenotypic uncertainty"

Vaibhav Mohanty, Anna Sappington, Eugene I. Shakhnovich, and Bonnie Berger

Appendices

Abbreviations used throughout the appendices include: GP (genotype-phenotype), deterministic genotype-phenotype (DGP) maps, and probabilistic genotype-phenotype (PrGP) maps.

A Derivation of the PrGP diffusion limit of population genetics as a stochastic differential equation

We derive an Itō stochastic differential equation for dynamics on probabilistic fitness landscapes with an arbitrary number of phenotypes and fitnesses. Here, fitness refers to growth rate given by average replication rate times number of offspring per unit time. Let G be the genotype set and let P be the phenotype set.

A.1 Selection and mutation upon replication, spontaneous mutation, and stochastic phenotype switching

At some time $t + \delta t$, we have

$$f_g^{(p)}(t+\delta t) = \frac{n_g^{(p)}(t+\delta t)}{\sum_{j\in P}\sum_{i\in G} n_i^{(j)}(t+\delta t)}. \tag{A.1}$$

Given fitness $X^{(p)}$ for a phenotype $p \in P$, we find that the number of individuals at time $t + \delta t$ which have at genotype $g \in G$ which map to phenotype p is given below, assuming δt is very small:

$$\begin{aligned} n_g^{(p)}(t+\delta t) = n_g^{(p)}(t) &+ \delta t \sum_{k\in P} n_g^{(k)}(t)\varphi_g^{(k\to p)} X^{(k)} \left(1 - \sum_{h\neq g}\mu_{g\to h}\right) \\ &+ \delta t \sum_{h\neq g}\sum_{k\in P} n_h^{(k)}(t)\varphi_g^{(k\to p)} X^{(k)}\mu_{h\to g} \\ &+ \delta t \sum_{h\neq g}\sum_{k\in P} n_h^{(k)}(t)\varphi_g^{(k\to p)} R^{(k)} m_{h\to g} \\ &- \delta t\, n_g^{(p)}(t) R^{(p)} \sum_{h\neq g} m_{g\to h} \\ &+ \delta t \sum_{k\neq p} n_g^{(k)}(t) S^{(k)}\sigma_g^{(k\to p)} \\ &- \delta t\, n_g^{(p)}(t) S^{(p)} \sum_{k\neq p}\sigma_g^{(p\to k)} \end{aligned} \tag{A.2}$$

where $\mu_{g\to h}$ is the probability that a new individual mutates from genotype g to genotype h at birth, $m_{g\to h}$ is the probability that an individual mutates from genotype g to genotype h spontaneously at a rate $R^{(p)}$ specified by the starting phenotype p, and $\sigma_g^{(p\to k)}$ is the probability that an individual changes its phenotype spontaneously from phenotype p to phenotype k at a rate $S^{(p)}$ specified by the by the starting phenotype p and the genotype g. Thus, we have additive contributions from growth of each phenotype, additive and subtractive flux from mutation events which take place at birth (which all depend on $\varphi_g^{(k\to p)}$ because phenotype assignment occurs at birth but may depend on the phenotype k of the parent), additive and subtractive flux from spontaneous mutations which may occur in already existing individuals (which may occur at a rate independent of any growth rates/fitnesses), and additive and subtractive flux from stochastic phenotype switching which may occur in already existing individuals (which also may occur at a rate independent of any growth rates/fitnesses).

bioRxiv preprint doi: https://doi.org/10.64898/2026.03.15.711953; this version posted March 16, 2026. The copyright holder for this preprint (which was not certified by peer review) is the author/funder, who has granted bioRxiv a license to display the preprint in perpetuity. It is made available under aCC-BY 4.0 International license.

We rewrite the equation above, grouping terms into each of these separate contributions

$$\begin{aligned} n_g^{(p)}(t+\delta t) = n_g^{(p)}(t) + \delta t \sum_{k\in P} n_g^{(k)}(t)\varphi_g^{(k\to p)} X^{(k)} \\ + \delta t \sum_{h\neq g}\sum_{k\in P} X^{(k)}\left[n_h^{(k)}(t)\varphi_g^{(k\to p)}\mu_{h\to g} - n_g^{(k)}(t)\mu_{g\to h}\right] \\ + \delta t \sum_{h\neq g}\sum_{k\in P} R^{(k)}\left[n_h^{(k)}(t)\varphi_g^{(k\to p)} m_{h\to g} - \delta_{pk} n_g^{(k)}(t) m_{g\to h}\right] \\ + \delta t \sum_{k\neq p}\left[S^{(k)} n_g^{(k)}(t)\sigma_g^{(k\to p)} - S^{(p)} n_g^{(p)}(t)\sigma_g^{(p\to k)}\right]. \end{aligned} \tag{A.3}$$

We now compute the denominator of the ratio in eq. (A.1):

$$\begin{aligned} \sum_{i\in G}\sum_{j\in P} n_i^{(j)}(t+\delta t) = \sum_{i\in G}\sum_{j\in P} n_i^{(j)}(t) + \delta t \sum_{i\in G}\sum_{j\in P}\sum_{k\in P} n_i^{(k)}(t)\varphi_i^{(k\to j)} X^{(k)} \\ + \delta t \sum_{i\in G}\sum_{j\in P}\sum_{h\neq i}\sum_{k\in P} X^{(k)}\left[n_h^{(k)}(t)\varphi_i^{(k\to j)}\mu_{h\to i} - n_i^{(k)}(t)\mu_{i\to h}\right] \\ + \delta t \sum_{i\in G}\sum_{j\in P}\sum_{h\neq i}\sum_{k\in P} R^{(k)}\left[n_h^{(k)}(t)\varphi_i^{(k\to j)} m_{h\to i} - \delta_{jk} n_i^{(k)}(t) m_{i\to h}\right] \\ + \delta t \sum_{i\in G}\sum_{j\in P}\sum_{k\neq j}\left[S^{(k)} n_i^{(k)}(t)\sigma^{(k\to j)} - S^{(j)} n_i^{(j)}(t)\sigma^{(j\to k)}\right]. \end{aligned} \tag{A.4}$$

First, we note that the first term normalizes

$$\sum_{i\in G}\sum_{j\in P} n_i^{(j)}(t) = N. \tag{A.5}$$

and

$$\sum_{j\in P}\varphi_i^{(k\to j)} = 1. \tag{A.6}$$

Next, we identify

$$\sum_{i\in G}\sum_{k\in P}\sum_{j\in P}\varphi_i^{(k\to j)} n_i^{(k)}(t) X^{(k)} = \sum_{i\in G}\sum_{k\in P} n_i^{(k)}(t) X^{(k)} = N\overline{X}(t) \tag{A.7}$$

as the mean fitness of the population as a function of time. The third term, which represents mutation upon replication, is simplified

$$\begin{aligned} \sum_{i\in G}\sum_{k\in P}\sum_{j\in P}\varphi_i^{(k\to j)}\sum_{h\neq i} X^{(k)}\left[n_h^{(k)}(t)\mu_{h\to i} - n_i^{(k)}(t)\mu_{i\to h}\right] \\ = \sum_{i\in G}\sum_{h\neq i}\sum_{k\in P} X^{(k)}\left[n_h^{(k)}(t)\mu_{h\to i} - n_i^{(k)}(t)\mu_{i\to h}\right]. \end{aligned} \tag{A.8}$$

due to the symmetry of the sum

$$\sum_{i\in G}\sum_{h\neq i}\alpha(i,h) = \sum_{h\in G}\sum_{i\neq h}\alpha(i,h), \tag{A.9}$$

we have that

$$\sum_{i\in G}\sum_{h\neq i} n_h^{(k)}(t)\mu_{h\to i} = \sum_{h\in G}\sum_{i\neq h} n_h^{(k)}(t)\mu_{h\to i} = \sum_{i\in G}\sum_{h\neq i} n_i^{(k)}(t)\mu_{i\to h}, \tag{A.10}$$

where in the second step we have swapped index labels h and i. Therefore, the term

$$\sum_{i\in G}\sum_{h\neq i}\sum_{k\in P} X^{(k)}\left[n_h^{(k)}(t)\mu_{h\to i} - n_i^{(k)}(t)\mu_{i\to h}\right] = 0. \tag{A.11}$$

bioRxiv preprint doi: https://doi.org/10.64898/2026.03.15.711953; this version posted March 16, 2026. The copyright holder for this preprint (which was not certified by peer review) is the author/funder, who has granted bioRxiv a license to display the preprint in perpetuity. It is made available under aCC-BY 4.0 International license.

The fourth term, representing spontaneous mutations, is given by

$$\sum_{i\in G}\sum_{j\in P}\sum_{h\neq i}\sum_{k\in P} R^{(k)}\left[\varphi_i^{(k\to j)} n_h^{(k)}(t) m_{h\to i} - \delta_{jk} n_i^{(k)}(t) m_{i\to h}\right] = \sum_{i\in G}\sum_{h\neq i}\sum_{k\in P} R^{(k)}\left[n_h^{(k)}(t) m_{h\to i} - n_i^{(k)}(t) m_{i\to h}\right]. \tag{A.12}$$

Once again, we have

$$\sum_{i\in G}\sum_{h\neq i} n_h^{(k)}(t) m_{h\to i} = \sum_{h\in G}\sum_{i\neq h} n_h^{(k)}(t) m_{h\to i} = \sum_{i\in G}\sum_{h\neq i} n_i^{(k)}(t) m_{i\to h}, \tag{A.13}$$

which means

$$\sum_{i\in G}\sum_{h\neq i}\sum_{k\in P} R^{(k)}\left[n_h^{(k)}(t) m_{h\to i} - n_i^{(k)}(t) m_{i\to h}\right] = 0. \tag{A.14}$$

The fifth term, representing stochastic phenotype switching, is

$$\sum_{i\in G}\sum_{j\in P}\sum_{k\neq j}\left[S^{(k)} n_i^{(k)}(t)\sigma^{(k\to j)} - S^{(j)} n_i^{(j)}(t)\sigma^{(j\to k)}\right]. \tag{A.15}$$

Using the same justification as before,

$$\sum_{j\in P}\sum_{k\neq j} S^{(k)} n_i^{(k)}(t)\sigma^{(k\to j)} = \sum_{k\in P}\sum_{j\neq k} S^{(k)} n_i^{(k)}(t)\sigma^{(k\to j)} = \sum_{j\in P}\sum_{k\neq j} S^{(j)} n_i^{(j)}(t)\sigma^{(j\to k)}, \tag{A.16}$$

which means

$$\sum_{i\in G}\sum_{j\in P}\sum_{k\neq j}\left[S^{(k)} n_i^{(k)}(t)\sigma^{(k\to j)} - S^{(j)} n_i^{(j)}(t)\sigma^{(j\to k)}\right] = 0. \tag{A.17}$$

Therefore, the denominator of eq. (A.1) is

$$\begin{aligned}
\sum_{i\in G}\sum_{j\in P} n_i^{(j)}(t+\delta t) &= \sum_{i\in G}\sum_{j\in P} n_i^{(j)}(t) + \delta t \sum_{i\in G}\sum_{k\in P}\sum_{j\in P}\varphi_i^{(k\to j)} n_i^{(k)}(t) X^{(k)} \\
&\quad + \delta t \sum_{i\in G}\sum_{k\in P}\sum_{j\in P}\varphi_i^{(k\to j)}\sum_{h\neq i} X^{(k)}\left[n_h^{(k)}(t)\mu_{h\to i} - n_i^{(k)}(t)\mu_{i\to h}\right] \\
&\quad + \delta t \sum_{i\in G}\sum_{j\in P}\sum_{h\neq i}\sum_{k\in P} R^{(k)}\left[\varphi_i^{(k\to j)} n_h^{(k)}(t) m_{h\to i} - \delta_{jk} n_i^{(k)}(t) m_{i\to h}\right] \\
&\quad + \delta t \sum_{i\in G}\sum_{j\in P}\sum_{k\neq j}\left[S^{(k)} n_i^{(k)}(t)\sigma^{(k\to j)} - S^{(j)} n_i^{(j)}(t)\sigma^{(j\to k)}\right] \\
&= N + N\delta t\, \overline{X}(t).
\end{aligned} \tag{A.18}$$

We can now expand eq. (A.1) to $\mathcal{O}(\delta t)$:

$$\begin{aligned}
f_g^{(p)}(t+\delta t) = \Bigg[& f_g^{(p)}(t) + \delta t \sum_{k\in P} f_g^{(k)}(t)\varphi_g^{(k\to p)} X^{(k)} \\
& + \delta t \sum_{h\neq g}\sum_{k\in P} X^{(k)}\left[f_h^{(k)}(t)\varphi_g^{(k\to p)}\mu_{h\to g} - f_g^{(k)}(t)\mu_{g\to h}\right] \\
& + \delta t \sum_{h\neq g}\sum_{k\in P} R^{(k)}\left[f_h^{(k)}(t)\varphi_g^{(k\to p)} m_{h\to g} - \delta_{pk} f_g^{(k)}(t) m_{g\to h}\right] \\
& + \delta t \sum_{k\neq p}\left[S^{(k)} f_g^{(k)}(t)\sigma_g^{(k\to p)} - S^{(p)} f_g^{(p)}(t)\sigma_g^{(p\to k)}\right]\Bigg]\left(1 - \delta t\, \overline{X}(t) + \mathcal{O}(\delta t^2)\right).
\end{aligned} \tag{A.19}$$

bioRxiv preprint doi: https://doi.org/10.64898/2026.03.15.711953; this version posted March 16, 2026. The copyright holder for this preprint (which was not certified by peer review) is the author/funder, who has granted bioRxiv a license to display the preprint in perpetuity. It is made available under aCC-BY 4.0 International license.

Distributing, we have

$$\begin{aligned} f_g^{(p)}(t+\delta t) = f_g^{(p)}(t) + \delta t \Bigg\{ & \Bigg[\sum_{k \in P} f_g^{(k)}(t) \varphi_g^{(k\to p)} X^{(k)} - f_g^{(p)}(t)\overline{X}(t) \Bigg] \\ & + \sum_{h \neq g} \sum_{k \in P} X^{(k)} \left[f_h^{(k)}(t) \varphi_g^{(k\to p)} \mu_{h\to g} - f_g^{(k)}(t) \mu_{g\to h} \right] \\ & + \sum_{h \neq g} \sum_{k \in P} R^{(k)} \left[f_h^{(k)}(t) \varphi_g^{(k\to p)} m_{h\to g} - \delta_{pk} f_g^{(k)}(t) m_{g\to h} \right] \\ & + \sum_{k \neq p} \left[S^{(k)} f_g^{(k)}(t) \sigma_g^{(k\to p)} - S^{(p)} f_g^{(p)}(t) \sigma_g^{(p\to k)} \right] \Bigg\} + \mathcal{O}(\delta t^2). \end{aligned} \tag{A.20}$$

Noting that

$$f_g^{(p)}(t+\delta t) = f_g^{(p)}(t) + \delta t \, \frac{\partial f_g^{(p)}}{\partial t} + \mathcal{O}(\delta t^2), \tag{A.21}$$

we can write the diffusion limit at infinite population size for dynamics on fitness landscapes with probabilistic genotype-phenotype (PrGP) mapping

$$\begin{aligned} \left(\frac{\partial f_g^{(p)}}{\partial t} \right)_{N\to\infty} = & \underbrace{\sum_{k \in P} f_g^{(k)}(t) \varphi_g^{(k\to p)} X^{(k)} - f_g^{(p)}(t)\overline{X}(t)}_{\text{PrGP selection}} \\ & + \underbrace{\sum_{h \neq g} \sum_{k \in P} \varphi_g^{(k\to p)} X^{(k)} \left[f_h^{(k)}(t) \mu_{h\to g} - f_g^{(k)}(t) \mu_{g\to h} \right]}_{\text{mutation upon replication}} \\ & + \underbrace{\sum_{h \neq g} \sum_{k \in P} R^{(k)} \left[f_h^{(k)}(t) \varphi_g^{(k\to p)} m_{h\to g} - \delta_{pk} f_g^{(k)}(t) m_{g\to h} \right]}_{\text{spontaneous mutation}} \\ & + \underbrace{\sum_{k \neq p} \left[S^{(k)} f_g^{(k)}(t) \sigma_g^{(k\to p)} - S^{(p)} f_g^{(p)}(t) \sigma_g^{(p\to k)} \right]}_{\text{stochastic phenotype switching}}. \end{aligned} \tag{A.22}$$

Notably, when $\varphi_g^{(k\to p)} = 1$ for only one phenotype p, meaning that $f_g^{(p)}$ is nonzero for only one phenotype, the exact diffusion limit of population genetics is recovered in the infinite population limit.

Now, we note that we can split the PrGP selection term to two terms resembling classical (DGP) selection (i.e. the replicator term), plus a fitness-dependent phenotype switching term which refers to phenotype noise during replication. To see this, we note

$$\begin{aligned} & \underbrace{\sum_{k \in P} f_g^{(k)}(t) \varphi_g^{(k\to p)} X^{(k)} - f_g^{(p)}(t)\overline{X}(t)}_{\text{PrGP selection}} \\ & = \sum_{k \in P} f_g^{(k)}(t) \varphi_g^{(k\to p)} X^{(k)} - f_g^{(p)}(t)\overline{X}(t) + f_g^{(p)} X^{(p)} - f_g^{(p)} X^{(p)} \\ & = f_g^{(p)}(t)(X^{(p)} - \overline{X}(t)) + \sum_{k \in P} f_g^{(k)}(t) \varphi_g^{(k\to p)} X^{(k)} - f_g^{(p)} X^{(p)} \\ & = f_g^{(p)}(t)(X^{(p)} - \overline{X}(t)) + \sum_{k \neq p} f_g^{(k)}(t) \varphi_g^{(k\to p)} X^{(k)} + f_g^{(p)}(t) \varphi_g^{(p\to p)} X^{(p)} - f_g^{(p)} X^{(p)} \\ & = f_g^{(p)}(t)(X^{(p)} - \overline{X}(t)) + \sum_{k \neq p} f_g^{(k)}(t) \varphi_g^{(k\to p)} X^{(k)} - f_g^{(p)}(t) X^{(p)} \left(1 - \varphi_g^{(p\to p)} \right) \\ & = f_g^{(p)}(t)(X^{(p)} - \overline{X}(t)) + \sum_{k \neq p} f_g^{(k)}(t) \varphi_g^{(k\to p)} X^{(k)} - f_g^{(p)}(t) X^{(p)} \sum_{k \neq p} \varphi_g^{(p\to k)} \\ & = \underbrace{f_g^{(p)}(t)(X^{(p)} - \overline{X}(t))}_{\text{classical (DGP) selection}} + \underbrace{\sum_{k \neq p} \left[X^{(k)} f_g^{(k)}(t) \varphi_g^{(k\to p)} - X^{(p)} f_g^{(p)}(t) \varphi_g^{(p\to k)} \right]}_{\text{phenotype noise at birth}} \end{aligned} \tag{A.23}$$

bioRxiv preprint doi: https://doi.org/10.64898/2026.03.15.711953; this version posted March 16, 2026. The copyright holder for this preprint (which was not certified by peer review) is the author/funder, who has granted bioRxiv a license to display the preprint in perpetuity. It is made available under aCC-BY 4.0 International license.

Finally, we can rewrite the diffusion limit at infinite population size as

$$\begin{aligned}\left(\frac{\partial f_g^{(p)}}{\partial t}\right)_{N\to\infty} &= \underbrace{f_g^{(p)}(t)(X^{(p)} - \overline{X}(t))}_{\text{classical (DGP) selection}} \\ &+ \underbrace{\sum_{h\neq g}\sum_{k\in P}\varphi_g^{(k\to p)}X^{(k)}\left[f_h^{(k)}(t)\mu_{h\to g} - f_g^{(k)}(t)\mu_{g\to h}\right]}_{\text{mutation upon replication}} \\ &+ \underbrace{\sum_{h\neq g}\sum_{k\in P}R^{(k)}\left[f_h^{(k)}(t)\varphi_g^{(k\to p)}m_{h\to g} - \delta_{pk}f_g^{(k)}(t)m_{g\to h}\right]}_{\text{spontaneous mutation}} \\ &+ \underbrace{\sum_{k\neq p}\left[X^{(k)}f_g^{(k)}(t)\varphi_g^{(k\to p)} - X^{(p)}f_g^{(p)}(t)\varphi_g^{(p\to k)}\right]}_{\text{phenotype noise at birth}} \\ &+ \underbrace{\sum_{k\neq p}\left[S^{(k)}f_g^{(k)}(t)\sigma_g^{(k\to p)} - S^{(p)}f_g^{(p)}(t)\sigma_g^{(p\to k)}\right]}_{\text{stochastic phenotype switching}}.\end{aligned} \tag{A.24}$$

A.2 Genetic drift

For finite populations of size N, evolutionary dynamics will be subject to genetic drift due to the sampling noise from generation to generation. Generally, in the standard derivation of the (deterministic) Kimura equation, one first assumes that there are no differences in fitness and no mutations at birth. However, as we will discuss in detail in the next session, even if all fitnesses are equal, the absolute fitness determines how many individuals are transferred over from one generation to the next (thus without any need for probabilistic phenotype selection) versus how many individuals are born, all of whom must be probabilistically assigned a phenotype at birth.

We now consider only selection and probabilistic phenotype determination at birth. Thus, the number of individuals with genotype g and phenotype p at $t+\delta t$ will be a random variable sampled from a Poisson distribution

$$n_g^{(p)}(t+\delta t) \sim \text{Poisson}\left(n_g^{(p)}(t) + \delta t\sum_{k\in P}n_g^{(k)}(t)\varphi_g^{(k\to p)}X^{(k)}\right). \tag{A.25}$$

We can write this in the normal distribution approximation

$$\begin{aligned}n_g^{(p)}(t+\delta t) \approx{}& n_g^{(p)}(t) + \delta t\sum_{k\in P}n_g^{(k)}(t)\varphi_g^{(k\to p)}X^{(k)} \\ &+ Z_g^{(p)}(t)\sqrt{n_g^{(p)}(t) + \delta t\sum_{k\in P}n_g^{(k)}(t)\varphi_g^{(k\to p)}X^{(k)}},\end{aligned} \tag{A.26}$$

where each $Z_g^{(p)}(t) \sim \mathcal{N}(0,1)$ is independent and normally distributed. At some time $t+\delta t$, we have

$$f_g^{(p)}(t+\delta t) = \frac{n_g^{(p)}(t+\delta t)}{\sum_{j\in P}\sum_{i\in G}n_i^{(j)}(t+\delta t)}. \tag{A.27}$$

bioRxiv preprint doi: https://doi.org/10.64898/2026.03.15.711953; this version posted March 16, 2026. The copyright holder for this preprint (which was not certified by peer review) is the author/funder, who has granted bioRxiv a license to display the preprint in perpetuity. It is made available under aCC-BY 4.0 International license.

Replacing with the approximation, we have

$$
\begin{aligned}
&f_g^{(p)}(t+\delta t) \approx \\
&\frac{n_g^{(p)}(t)+\delta t\ \sum_{k\in P} n_g^{(k)}(t)\varphi_g^{(k\to p)}X^{(k)}+Z_g^{(p)}(t)\sqrt{n_g^{(p)}(t)+\delta t\ \sum_{k\in P} n_g^{(k)}(t)\varphi_g^{(k\to p)}X^{(k)}}}{\sum_{j\in P}\sum_{i\in G}\left[n_i^{(j)}(t)+\delta t\ \sum_{k\in P} n_i^{(k)}(t)\varphi_i^{(k\to j)}X^{(k)}+Z_i^{(j)}(t)\sqrt{n_i^{(j)}(t)+\delta t\ \sum_{k\in P} n_i^{(k)}(t)\varphi_i^{(k\to j)}X^{(k)}}\right]} \\
&=\frac{n_g^{(p)}(t)+\delta t\ \sum_{k\in P} n_g^{(k)}(t)\varphi_g^{(k\to p)}X^{(k)}+Z_g^{(p)}(t)\sqrt{n_g^{(p)}(t)+\delta t\ \sum_{k\in P} n_g^{(k)}(t)\varphi_g^{(k\to p)}X^{(k)}}}{N+\delta t\ N\overline{X}(t)+\sum_{j\in P}\sum_{i\in G} Z_i^{(j)}(t)\sqrt{n_i^{(j)}(t)+\delta t\ \sum_{k\in P} n_i^{(k)}(t)\varphi_i^{(k\to j)}X^{(k)}}} \\
&=\frac{f_g^{(p)}(t)+\delta t\ \sum_{k\in P} f_g^{(k)}(t)\varphi_g^{(k\to p)}X^{(k)}+\frac{1}{\sqrt{N}}Z_g^{(p)}(t)\sqrt{f_g^{(p)}(t)+\delta t\ \sum_{k\in P} f_g^{(k)}(t)\varphi_g^{(k\to p)}X^{(k)}}}{1+\delta t\ \overline{X}(t)+\sum_{j\in P}\sum_{i\in G}\frac{1}{\sqrt{N}}Z_i^{(j)}(t)\sqrt{f_i^{(j)}(t)+\delta t\ \sum_{k\in P} f_i^{(k)}(t)\varphi_i^{(k\to j)}X^{(k)}}}.
\end{aligned}
\tag{A.28}
$$

The standard Gaussian $Z_g^{(p)}(t)$ can be related to white noise $\eta_g^{(p)}(t)$ as $Z_g^{(p)}(t) = \sqrt{\delta t}\eta_g^{(p)}(t)$ because $Z_g^{(p)}(t)$ has a variance of 1 over a timestep δt. The white noise has mean $\langle \eta_g^{(p)}(t)\rangle = 0$ and correlation $\langle \eta_g^{(p)}(t)\eta_{g'}^{(p')}(t')\rangle = \delta_{gg'}\delta_{pp'}\delta(t-t')$. It now follows that

$$
\begin{aligned}
&f_g^{(p)}(t+\delta t) \approx \\
&\frac{f_g^{(p)}(t)+\delta t\ \sum_{k\in P} f_g^{(k)}(t)\varphi_g^{(k\to p)}X^{(k)}+\frac{1}{\sqrt{N}}\eta_g^{(p)}(t)\sqrt{\delta t}\sqrt{f_g^{(p)}(t)+\delta t\ \sum_{k\in P} f_g^{(k)}(t)\varphi_g^{(k\to p)}X^{(k)}}}{1+\delta t\ \overline{X}(t)+\sum_{j\in P}\sum_{i\in G}\frac{1}{\sqrt{N}}\eta_i^{(j)}(t)\sqrt{\delta t}\sqrt{f_i^{(j)}(t)+\delta t\ \sum_{k\in P} f_i^{(k)}(t)\varphi_i^{(k\to j)}X^{(k)}}}.
\end{aligned}
\tag{A.29}
$$

Taylor expanding for small δt, we have

$$
\begin{aligned}
f_g^{(p)}(t+\delta t) \approx f_g^{(p)}(t) &+ \sqrt{\delta t}\left[\eta_g^{(p)}(t)\sqrt{\frac{f_g^{(p)}(t)}{N}} - f_g^{(p)}(t)\sum_{j\in P}\sum_{i\in G}\eta_i^{(j)}(t)\sqrt{\frac{f_i^{(j)}(t)}{N}}\right] \\
&+\delta t\left[\sum_{k\in P} f_g^{(k)}(t)\varphi_g^{(k\to p)}X^{(k)} - f_g^{(p)}(t)\overline{X}(t) - \eta_g^{(p)}(t)\frac{\sqrt{f_g^{(p)}(t)}}{N}\sum_{j\in P}\sum_{i\in G}\eta_i^{(j)}(t)\sqrt{f_i^{(j)}(t)}\right. \\
&\left.+\frac{f_g^{(p)}(t)}{N}\left(\sum_{j\in P}\sum_{i\in G}\eta_i^{(j)}(t)\sqrt{f_i^{(j)}(t)}\right)^2\right].
\end{aligned}
\tag{A.30}
$$

Taking the diffusion limit of small δt and large N, we ignore the sub-leading order terms which are $\mathcal{O}(1/N)$, retaining only the terms which are $\mathcal{O}(1)$ and $\mathcal{O}(1/\sqrt{N})$ in N:

$$
\begin{aligned}
f_g^{(p)}(t+\delta t) \approx f_g^{(p)}(t) &+ \sqrt{\delta t}\left[\eta_g^{(p)}(t)\sqrt{\frac{f_g^{(p)}(t)}{N}} - f_g^{(p)}(t)\sum_{j\in P}\sum_{i\in G}\eta_i^{(j)}(t)\sqrt{\frac{f_i^{(j)}(t)}{N}}\right] \\
&+\delta t\left[\sum_{k\in P} f_g^{(k)}(t)\varphi_g^{(k\to p)}X^{(k)} - f_g^{(p)}(t)\overline{X}(t)\right].
\end{aligned}
\tag{A.31}
$$

Rearranging, we have

$$
\begin{aligned}
\frac{f_g^{(p)}(t+\delta t)-f_g^{(p)}(t)}{\delta t} \approx \frac{1}{\sqrt{N\delta t}} &\left[\sum_{j\in P}\sum_{i\in G}\left(\delta_{gi}\delta_{pj}-f_g^{(p)}\right)\eta_i^{(j)}(t)\sqrt{f_i^{(j)}(t)}\right] \\
&+\sum_{k\in P} f_g^{(k)}(t)\varphi_g^{(k\to p)}X^{(k)} - f_g^{(p)}(t)\overline{X}(t).
\end{aligned}
\tag{A.32}
$$

We note that the persistence of $1/\sqrt{\delta t}$ also cancels the units of the white noise, which, from the correlation $\langle \eta_g^{(p)}(t)\eta_{g'}^{(p')}(t')\rangle = \delta_{gg'}\delta_{pp'}\delta(t-t')$ indicates that $\eta_g^{(p)}(t) \sim [\text{time}]^{-1/2}$. Since we are using δt to represent

bioRxiv preprint doi: https://doi.org/10.64898/2026.03.15.711953; this version posted March 16, 2026. The copyright holder for this preprint (which was not certified by peer review) is the author/funder, who has granted bioRxiv a license to display the preprint in perpetuity. It is made available under aCC-BY 4.0 International license.

physical time (instead of a non-dimensional time unit such as generations), we let $\widetilde{N} \equiv N\delta t$ represent a scaled population size which has been multiplied by the generation time[84]. We now have, in the diffusion limit, the selection term and the genetic drift term:

$$\begin{aligned}\frac{\partial f_g^{(p)}}{\partial t} \approx & \underbrace{\frac{1}{\sqrt{\widetilde{N}}} \sum_{j\in P}\sum_{i\in G} \left(\delta_{gi}\delta_{pj} - f_g^{(p)}\right) \eta_i^{(j)}(t)\sqrt{f_i^{(j)}(t)}}_{\text{genetic drift}} \\ & + \underbrace{\sum_{k\in P} f_g^{(k)}(t)\varphi_g^{(k\to p)} X^{(k)} - f_g^{(p)}(t)\overline{X}(t)}_{\text{PrGP selection}}.\end{aligned} \tag{A.33}$$

The selection term matches the one derived in eq. (A.24), and the drift term is the same as the diffusion limit of population genetics in the deterministic limit. Thus, we can now write the Itō stochastic differential equation in the Langevin form in the finite population limit, for generation time-scaled population $\widetilde{N}$:

$$\begin{aligned}\frac{\partial f_g^{(p)}}{\partial t} = & \underbrace{f_g^{(p)}(t)(X^{(p)} - \overline{X}(t))}_{\text{classical (DGP) selection}} \\ & + \underbrace{\sum_{h\neq g}\sum_{k\in P} \varphi_g^{(k\to p)} X^{(k)} \left[f_h^{(k)}(t)\mu_{h\to g} - f_g^{(k)}(t)\mu_{g\to h}\right]}_{\text{mutation upon replication}} \\ & + \underbrace{\sum_{h\neq g}\sum_{k\in P} R^{(k)} \left[f_h^{(k)}(t)\varphi_g^{(k\to p)} m_{h\to g} - \delta_{pk} f_g^{(k)}(t) m_{g\to h}\right]}_{\text{spontaneous mutation}} \\ & + \underbrace{\sum_{k\neq p} \left[X^{(k)} f_g^{(k)}(t)\varphi_g^{(k\to p)} - X^{(p)} f_g^{(p)}(t)\varphi_g^{(p\to k)}\right]}_{\text{phenotype noise at birth}} \\ & + \underbrace{\sum_{k\neq p} \left[S^{(k)} f_g^{(k)}(t)\sigma_g^{(k\to p)} - S^{(p)} f_g^{(p)}(t)\sigma_g^{(p\to k)}\right]}_{\text{stochastic phenotype switching}} \\ & + \underbrace{\frac{1}{\sqrt{\widetilde{N}}} \sum_{j\in P}\sum_{i\in G} \left(\delta_{gi}\delta_{pj} - f_g^{(p)}\right) \eta_i^{(j)}(t)\sqrt{f_i^{(j)}(t)}}_{\text{genetic drift}}.\end{aligned} \tag{A.34}$$

A.3 Consistency check: recovering the classical diffusion limit of population genetics in the deterministic genotype-phenotype map regime

We now ask if the deterministic genotype-phenotype (DGP) map—which is the classical diffusion limit of population genetics, equivalent to the Langevin formulation of the multilocus Kimura equation—can be recovered the PrGP diffusion limit which we have just derived. In a standard treatment of population genetics, we would typically only consider selection, mutations, and genetic drift—and maybe recombination, but we are not considering recombination in this treatment. Here, we include both

bioRxiv preprint doi: https://doi.org/10.64898/2026.03.15.711953; this version posted March 16, 2026. The copyright holder for this preprint (which was not certified by peer review) is the author/funder, who has granted bioRxiv a license to display the preprint in perpetuity. It is made available under aCC-BY 4.0 International license.

mutations upon replication as well as spontaneous mutations.

$$\begin{aligned}\frac{\partial f_g^{(p)}}{\partial t} &= \underbrace{\sum_{k\in P} f_g^{(k)}(t)\varphi_g^{(k\to p)}X^{(k)} - f_g^{(p)}(t)\overline{X}(t)}_{\text{PrGP selection}} \\ &\quad + \underbrace{\sum_{h\neq g}\sum_{k\in P} X^{(k)}\left[f_h^{(k)}(t)\varphi_g^{(k\to p)}\mu_{h\to g} - f_g^{(k)}(t)\mu_{g\to h}\right]}_{\text{mutation upon replication}} \\ &\quad + \underbrace{\sum_{h\neq g}\sum_{k\in P} R^{(k)}\left[f_h^{(k)}(t)\varphi_g^{(k\to p)}m_{h\to g} - \delta_{pk}f_g^{(k)}(t)m_{g\to h}\right]}_{\text{spontaneous mutation}} \\ &\quad + \underbrace{\frac{1}{\sqrt{\widetilde{N}}}\sum_{j\in P}\sum_{i\in G}\left(\delta_{gi}\delta_{pj} - f_g^{(p)}\right)\eta_i^{(j)}(t)\sqrt{f_i^{(j)}(t)}}_{\text{genetic drift}}.\end{aligned} \tag{A.35}$$

Now, we explore the deterministic genotype-phenotype mapping limit. For convenience, we set phenotype "g" to be the phenotype to which genotype g maps. This means that each frequency would only have one label: $f_g^{(p)}(t) \mapsto f_g^{(p)}(t)\delta_{gp}$, each genotype would have its own fitness $X^{(p)} \mapsto X^{(p)}\delta_{gp}$, and each probability would be a Kronecker delta that is independent of k such that$\varphi_g^{(k\to p)} \mapsto \delta_{pg}$. Making these replacements, we have

$$\begin{aligned}\frac{\partial (f_g^{(p)}\delta_{gp})}{\partial t} &= \delta_{gp}\sum_{k\in P}\delta_{gk}f_g^{(k)}(t)X^{(k)} - \delta_{gp}f_g^{(p)}(t)\overline{X}(t) \\ &\quad + \delta_{gp}\sum_{h\neq g}\sum_{k\in P}X^{(k)}\left[\delta_{hk}f_h^{(k)}(t)\mu_{h\to g} - \delta_{gk}f_g^{(k)}(t)\mu_{g\to h}\right] \\ &\quad + \sum_{h\neq g}\sum_{k\in P}R^{(k)}\left[\delta_{gp}\delta_{hk}f_h^{(k)}(t)m_{h\to g} - \delta_{pk}\delta_{gk}f_g^{(k)}(t)m_{g\to h}\right] \\ &\quad + \frac{1}{\sqrt{\widetilde{N}}}\sum_{j\in P}\sum_{i\in G}\left(\delta_{gi}\delta_{pj} - \delta_{gp}f_g^{(p)}\right)\eta_i^{(j)}(t)\sqrt{\delta_{ij}f_i^{(j)}(t)} \\ &= \delta_{gp}f_g^{(g)}(t)X^{(g)} - \delta_{gp}f_g^{(p)}(t)\overline{X}(t) \\ &\quad + \delta_{gp}\sum_{h\neq g}\left[X^{(h)}f_h^{(h)}(t)\mu_{h\to g} - X^{(g)}f_g^{(g)}(t)\mu_{g\to h}\right] \\ &\quad + \sum_{h\neq g}\left[\delta_{gp}R^{(h)}f_h^{(h)}(t)m_{h\to g} - \delta_{gp}R^{(p)}f_g^{(p)}(t)m_{g\to h}\right] \\ &\quad + \frac{1}{\sqrt{\widetilde{N}}}\left[\eta_g^{(p)}(t)\delta_{gp}\sum_{j\in P}\sqrt{\delta_{gj}f_g^{(j)}(t)} - \delta_{gp}f_g^{(p)}(t)\sum_{i\in G}\eta_i^{(i)}\sqrt{f_i^{(i)}}\right] \\ &= \delta_{gp}f_g^{(g)}(t)X^{(g)} - \delta_{gp}f_g^{(p)}(t)\overline{X}(t) \\ &\quad + \delta_{gp}\sum_{h\neq g}\left[X^{(h)}f_h^{(h)}(t)\mu_{h\to g} - X^{(g)}f_g^{(g)}(t)\mu_{g\to h}\right] \\ &\quad + \delta_{gp}\sum_{h\neq g}\left[R^{(h)}f_h^{(h)}(t)m_{h\to g} - R^{(p)}f_g^{(p)}(t)m_{g\to h}\right] \\ &\quad + \delta_{gp}\frac{1}{\sqrt{\widetilde{N}}}\left[\eta_g^{(p)}(t)\sqrt{f_g^{(g)}(t)} - f_g^{(p)}(t)\sum_{i\in G}\eta_i^{(i)}\sqrt{f_i^{(i)}}\right].\end{aligned} \tag{A.36}$$

bioRxiv preprint doi: https://doi.org/10.64898/2026.03.15.711953; this version posted March 16, 2026. The copyright holder for this preprint (which was not certified by peer review) is the author/funder, who has granted bioRxiv a license to display the preprint in perpetuity. It is made available under aCC-BY 4.0 International license.

Summing over all phenotypes p, and relabeling $f_g^{(g)}(t) \equiv f_g$, $\eta_g^{(g)}(t) \equiv \eta_g(t)$, $R^{(g)} = R_g$, and $X^{(g)} = X_g$ we have

$$\begin{aligned}
\sum_{p\in P} \delta_{gp} \frac{\partial f_g^{(p)}}{\partial t} = \frac{\partial f_g}{\partial t} &= \sum_{p\in P} \left[\delta_{gp} f_g^{(g)}(t) X^{(g)} - \delta_{gp} f_g^{(p)}(t)\overline{X}(t)\right] \\
&\quad + \sum_{p\in P} \delta_{gp} \sum_{h\neq g} \left[X^{(h)} f_h^{(h)}(t)\mu_{h\to g} - X^{(g)} f_g^{(g)}(t)\mu_{g\to h}\right] \\
&\quad + \sum_{p\in P} \delta_{gp} \sum_{h\neq g} \left[R^{(h)} f_h^{(h)}(t) m_{h\to g} - R^{(p)} f_g^{(p)}(t) m_{g\to h}\right] \\
&\quad + \sum_{p\in P} \delta_{gp} \frac{1}{\sqrt{\widetilde{N}}} \left[\eta_g^{(p)}(t)\sqrt{f_g^{(g)}(t)} - f_g^{(p)}(t) \sum_{i\in G} \eta_i^{(i)} \sqrt{f_i^{(i)}}\right] \\
&= f_g(t) X_g - f_g(t)\overline{X}(t) \\
&\quad + \sum_{h\neq g} \left[X^{(p)} f_h(t)\mu_{h\to g} - X_g f_g(t)\mu_{g\to h}\right] \\
&\quad + \sum_{h\neq g} \left[R_h f_h^{(h)}(t) m_{h\to g} - R_g f_g(t) m_{g\to h}\right] \\
&\quad + \frac{1}{\sqrt{\widetilde{N}}} \left[\eta_g(t)\sqrt{f_g(t)} - f_g \sum_{i\in G} \eta_i^{(i)} \sqrt{f_i^{(i)}}\right] \\
&= f_g(t)(X_g - \overline{X}(t)) \\
&\quad + \sum_{h\neq g} \left[f_h(t)(X^{(p)}\mu_{h\to g} + R_h m_{h\to g}) - f_g(t)(X_g \mu_{g\to h} + R_g m_{g\to h})\right] \\
&\quad + \frac{1}{\sqrt{\widetilde{N}}} \sum_{i\in G} (\delta_{gi} - f_g)\,\eta_i(t)\sqrt{f_i(t)}.
\end{aligned} \tag{A.37}$$

In the final step, we note that $X^{(p)}\mu_{h\to g} + R_h m_{h\to g}$ and $X_g \mu_{g\to h} + R_g m_{g\to h}$ are, respectively, the aggregate incoming and outgoing mutation *rates* which include both mutations upon replication and spontaneous mutations. We have thus arrived at the classical diffusion limit of population genetics, which is equivalent to the multilocus Kimura equation.

B Probabilistic serial dilution algorithm

Below, we provide pseudocode for the `ProSeD` algorithm, which enables evolutionary dynamics simulations in the presence of phenotype uncertainty.

C Phenotypic buoys

We now develop the theory of *phenotypic buoys*, where high fitness, low probability phenotypes can support (or "buoy") the prevalence of low fitness phenotypes to disproportionately high levels at long times.

C.1 Calculating equilibrium distributions

We first start with the full PrGP diffusion limit, in eq. (C.1), in the infinite population limit (zero genetic drift), and working in the regime where all mutations happen at birth (and not spontaneously), and there is no stochastic phenotype switching. At long times, the time derivatives of the frequencies will also go to zero, so we have

$$0 = \underbrace{\varphi_g^{(p)} \sum_{k\in P} f_g^{(k)} X^{(k)} - f_g^{(p)}(t)\overline{X}(t)}_{\text{PrGP selection}} + \underbrace{\varphi_g^{(p)} \sum_{h\neq g} \sum_{k\in P} X^{(k)} \left[f_h^{(k)} \mu_{h\to g} - f_g^{(k)}(t)\mu_{g\to h}\right]}_{\text{mutation upon replication}}. \tag{C.1}$$

bioRxiv preprint doi: https://doi.org/10.64898/2026.03.15.711953; this version posted March 16, 2026. The copyright holder for this preprint (which was not certified by peer review) is the author/funder, who has granted bioRxiv a license to display the preprint in perpetuity. It is made available under aCC-BY 4.0 International license.

Algorithm 1 Probabilistic Serial Dilution (ProSeD)

Require: Parameters $\{V, Q, N, T, c, \mu\}$, adjacency matrix A, phenotype probabilities π, reproduction probabilities r

procedure Initialize
  Generate or load genotype graph A
  Assign genotype-to-phenotype probabilities π
  Assign phenotype reproduction probabilities r
  Sample initial population Γ of size N from allowed (g, p) pairs
  Initialize frequency tensor $f[g, p, t] \leftarrow 0$
end procedure
procedure Simulate
  for $t \leftarrow 1$ to T **do**
    for each generation **do**
      Reproduction: offspring produced with prob. r_p and multiplicity c
      Mutation: offspring genotypes mutate with prob. μ via neighbors in A
      Phenotype assignment: offspring genotypes mapped to phenotypes via π
      Update population Γ
    end for
    Downsample Γ to size N
    Record frequencies $f[g, p, t]$
  end for
end procedure

This can be rearranged to

$$\overline{X}_{\text{eq}} f_g^{(p)}(t) = \varphi_g^{(p)} \sum_{k \in P} f_g^{(k)} X^{(k)} + \varphi_g^{(p)} \sum_{h \neq g} \sum_{k \in P} X^{(k)} \left[f_h^{(k)} \mu_{h \to g} - f_g^{(k)} \mu_{g \to h} \right]. \tag{C.2}$$

The above equation can be cast as an eigenvalue equation of the form

$$\overline{X}_{\text{eq}} \mathbf{f}_{\text{eq}} = H \mathbf{f}_{\text{eq}}, \tag{C.3}$$

where H is a matrix dependent on the fitnesses, PrGP map probabilities, and mutation probabilities. It can be shown using the Perron-Frobenius theorem that there exists a unique equilibrium frequency vector $\mathbf{f}_{\text{eq}}$ with principal eigenvalue equal to the equilibrium mean fitness $\overline{X}$. We now use this approach to analytically understand what we describe as a "phenotypic buoy," where a low-fitness, low-probability GP pair can still persist at equilibrium at unexpectedly high frequencies because it is "buoyed" by a high-fitness phenotype which has the same genotype. To do so, we explicitly construct the matrix form of the above equation for the 2 genotype, 2 phenotype case, which is one of the simplest possible examples that would exhibit this phenomenon.

C.2 Exactly analytically tractable example case: phenotypic buoy with 2 genotypes and 2 phenotypes

Suppose we haves two genotypes labeled 0 and 1, and two phenotypes labeled 0 and 1. We can then use the equations above to write the equilibrium matrix relation

$$\overline{X}_{\text{eq}} \begin{pmatrix} f_0^{(0)} \\ f_0^{(1)} \\ f_1^{(0)} \\ f_1^{(1)} \end{pmatrix} = \begin{pmatrix} \varphi_0^{(0)} X^{(0)}(1-\mu) & \varphi_0^{(0)} X^{(1)}(1-\mu) & \varphi_0^{(0)} X^{(0)}\mu & \varphi_0^{(0)} X^{(1)}\mu \\ \varphi_0^{(1)} X^{(0)}(1-\mu) & \varphi_0^{(1)} X^{(1)}(1-\mu) & \varphi_0^{(1)} X^{(0)}\mu & \varphi_0^{(1)} X^{(1)}\mu \\ \varphi_1^{(0)} X^{(0)}\mu & \varphi_1^{(0)} X^{(1)}\mu & \varphi_1^{(0)} X^{(0)}(1-\mu) & \varphi_1^{(0)} X^{(1)}(1-\mu) \\ \varphi_1^{(1)} X^{(0)}\mu & \varphi_1^{(1)} X^{(1)}\mu & \varphi_1^{(1)} X^{(0)}(1-\mu) & \varphi_1^{(1)} X^{(1)}(1-\mu) \end{pmatrix} \begin{pmatrix} f_0^{(0)} \\ f_0^{(1)} \\ f_1^{(0)} \\ f_1^{(1)} \end{pmatrix}, \tag{C.4}$$

where we have assumed uniform mutation probabilities μ. We can write the equilibrium equation in block matrix form

$$\overline{X}_{\text{eq}} \begin{pmatrix} \mathbf{f}_0 \\ \mathbf{f}_1 \end{pmatrix} = \begin{pmatrix} Y_0(1-\mu) & Y_0\mu \\ Y_1\mu & Y_1(1-\mu) \end{pmatrix} \begin{pmatrix} \mathbf{f}_0 \\ \mathbf{f}_1 \end{pmatrix}, \tag{C.5}$$

bioRxiv preprint doi: https://doi.org/10.64898/2026.03.15.711953; this version posted March 16, 2026. The copyright holder for this preprint (which was not certified by peer review) is the author/funder, who has granted bioRxiv a license to display the preprint in perpetuity. It is made available under aCC-BY 4.0 International license.

where we defined

$$\mathbf{f}_g = \begin{pmatrix} f_g^{(0)} \\ f_g^{(1)} \end{pmatrix} \tag{C.6}$$

and we can express the matrices as outer product

$$Y_g = \begin{pmatrix} \varphi_g^{(0)} \\ \varphi_g^{(1)} \end{pmatrix} \begin{pmatrix} X^{(0)} & X^{(1)} \end{pmatrix} \tag{C.7}$$

Next, we note that since we only have two phenotypes, we can further denote

$$\varphi_g^{(0)} = \pi_g \quad \text{and} \quad \varphi_g^{(1)} = (1 - \pi_g), \tag{C.8}$$

so that we have

$$Y_g = \begin{pmatrix} \pi_g \\ 1 - \pi_g \end{pmatrix} \begin{pmatrix} X^{(0)} & X^{(1)} \end{pmatrix} \equiv \mathbf{p}_g \mathbf{X}^T, \tag{C.9}$$

where we have defined vectors

$$\mathbf{p}_g = \begin{pmatrix} \pi_g \\ 1 - \pi_g \end{pmatrix} \quad \text{and} \quad \mathbf{X} = \begin{pmatrix} X^{(0)} \\ X^{(1)} \end{pmatrix}. \tag{C.10}$$

The eigenvalue equation can now be decomposed into two equations:

$$\begin{aligned} \overline{X}_{\text{eq}} \mathbf{f}_0 &= (1 - \mu) Y_0 \mathbf{f}_0 + \mu Y_0 \mathbf{f}_1, \quad \text{and} \\ \overline{X}_{\text{eq}} \mathbf{f}_1 &= \mu Y_1 \mathbf{f}_0 + (1 - \mu) Y_1 \mathbf{f}_1. \end{aligned} \tag{C.11}$$

In terms of the outer product decomposition, we can write

$$\begin{aligned} \overline{X}_{\text{eq}} \mathbf{f}_0 &= (1 - \mu) \mathbf{p}_0 \mathbf{X}^T \mathbf{f}_0 + \mu \mathbf{p}_0 \mathbf{X}^T \mathbf{f}_1, \quad \text{and} \\ \overline{X}_{\text{eq}} \mathbf{f}_1 &= \mu \mathbf{p}_1 \mathbf{X}^T \mathbf{f}_0 + (1 - \mu) \mathbf{p}_1 \mathbf{X}^T \mathbf{f}_1. \end{aligned} \tag{C.12}$$

Defining scalar dot products between the fitness and genotype-specific frequency vectors,

$$S_g \equiv \mathbf{X}^T \mathbf{f}_g = f_g^{(0)} X^{(0)} + f_g^{(1)} X^{(1)}, \tag{C.13}$$

we can write

$$\begin{aligned} \overline{X}_{\text{eq}} \mathbf{f}_0 &= (1 - \mu) \mathbf{p}_0 S_0 + \mu \mathbf{p}_0 S_1, \quad \text{and} \\ \overline{X}_{\text{eq}} \mathbf{f}_1 &= \mu \mathbf{p}_1 S_0 + (1 - \mu) \mathbf{p}_1 S_1. \end{aligned} \tag{C.14}$$

Multiplying both sides by $\mathbf{X}^T$ from the left, we have

$$\begin{aligned} \overline{X}_{\text{eq}} S_0 &= \alpha_0 \left[(1 - \mu) S_0 + \mu S_1 \right], \quad \text{and} \\ \overline{X}_{\text{eq}} S_1 &= \alpha_1 \left[\mu S_0 + (1 - \mu) S_1 \right], \end{aligned} \tag{C.15}$$

where we have also defined two new scalars which are dot products between the fitness and the genotype-specific GP mapping probability vectors:

$$\alpha_g \equiv \mathbf{X}^T \mathbf{p}_g = \pi_g X^{(0)} + (1 - \pi_g) X^{(1)}. \tag{C.16}$$

Rearranging the matrix equation, we can now frame the system of equations a new 2×2 matrix eigenvalue problem

$$\begin{pmatrix} \alpha_0 (1 - \mu) - \overline{X}_{\text{eq}} & \alpha_0 \mu \\ \alpha_1 \mu & \alpha_1 (1 - \mu) - \overline{X}_{\text{eq}} \end{pmatrix} \begin{pmatrix} S_0 \\ S_1 \end{pmatrix} = \mathbf{0}, \tag{C.17}$$

which holds when

$$\begin{aligned} 0 &= \det \begin{pmatrix} \alpha_0 (1 - \mu) - \overline{X}_{\text{eq}} & \alpha_0 \mu \\ \alpha_1 \mu & \alpha_1 (1 - \mu) - \overline{X}_{\text{eq}} \end{pmatrix} \\ &= \left(\alpha_0 (1 - \mu) - \overline{X}_{\text{eq}} \right) \left(\alpha_1 (1 - \mu) - \overline{X}_{\text{eq}} \right) - \alpha_0 \alpha_1 \mu^2. \end{aligned} \tag{C.18}$$

bioRxiv preprint doi: https://doi.org/10.64898/2026.03.15.711953; this version posted March 16, 2026. The copyright holder for this preprint (which was not certified by peer review) is the author/funder, who has granted bioRxiv a license to display the preprint in perpetuity. It is made available under aCC-BY 4.0 International license.

The original 4×4 matrix has two eigenvalues which are zero, because the block matrices are each rank-1 (since they are expressible as an outer product). The above quadratic equation provides the two non-zero eigenvalues and corresponding eigenvectors. Solving the quadratic equation, we obtain eigenvalues

$$\overline{X}_{\text{eq}} = \frac{(1-\mu)(\alpha_0+\alpha_1) \pm \sqrt{(1-\mu)^2(\alpha_0+\alpha_1)^2 - 4\alpha_0\alpha_1(1-2\mu)}}{2}. \tag{C.19}$$

As mentioned previously, the Perron-Frobenius theorem indicates that the principal eigenvalue and its corresponding eigenvector will give us the equilibrium mean fitness and frequency vector, respectively. Thus, we only take the positive solution:

$$\overline{X}_{\text{eq}} = \frac{(1-\mu)(\alpha_0+\alpha_1) + \sqrt{(1-\mu)^2(\alpha_0+\alpha_1)^2 - 4\alpha_0\alpha_1(1-2\mu)}}{2}, \tag{C.20}$$

to be the true equilibrium mean fitness. Now, we can use it to find the equilibrium frequency vector. First, assuming mutation probabilities are nonzero ($\mu > 0$), we note that

$$S_0 = \alpha_0\mu \quad \text{and} \quad S_1 = \overline{X}_{\text{eq}} - \alpha_0(1-\mu) \tag{C.21}$$

is a valid solution to eq. (C.17). Substituting these values into eq. (C.14), we can obtain unnormalized components of the equilibrium eigenvector

$$\begin{aligned} \mathbf{f}_0 &\propto \left[\alpha_0\mu(1-\mu) + \mu(\overline{X}_{\text{eq}} - \alpha_0(1-\mu))\right]\mathbf{p}_0 \\ \mathbf{f}_1 &\propto \left[\alpha_0\mu^2 + (1-\mu)(\overline{X}_{\text{eq}} - \alpha_0(1-\mu))\right]\mathbf{p}_1. \end{aligned} \tag{C.22}$$

Normalizing and simplifying the frequency vector, we have the complete equilibrium frequency vector expressed as

$$\mathbf{f}_{\text{eq}} = \frac{1}{1+\frac{\alpha_0(2\mu-1)}{\overline{X}_{\text{eq}}}} \begin{pmatrix} \mu\pi_0 \\ \mu(1-\pi_0) \\ \left[(1-\mu) + \frac{\alpha_0(2\mu-1)}{\overline{X}_{\text{eq}}}\right]\pi_1 \\ \left[(1-\mu) + \frac{\alpha_0(2\mu-1)}{\overline{X}_{\text{eq}}}\right](1-\pi_1) \end{pmatrix}. \tag{C.23}$$

This holds in general when the mutation rate μ is nonzero.

Limiting case: zero mutations ($\mu = 0$). When no mutations are present, the eigenspace becomes degenerate

$$\begin{aligned} \overline{X}_{\text{eq}} &= \frac{(\alpha_0+\alpha_1) \pm \sqrt{(\alpha_0+\alpha_1)^2 - 4\alpha_0\alpha_1}}{2} \\ &= \frac{(\alpha_0+\alpha_1) \pm \sqrt{\alpha_0^2+\alpha_1^2 - 2\alpha_0\alpha_1}}{2} \\ &= \frac{(\alpha_0+\alpha_1) \pm |\alpha_0-\alpha_1|}{2}. \end{aligned} \tag{C.24}$$

When $\alpha_0 > \alpha_1$,

$$\overline{X}_{\text{eq}} = \alpha_0, \tag{C.25}$$

from which it follows that

$$\begin{aligned} \mathbf{f}_0 &\propto \mathbf{p}_0 \\ \mathbf{f}_1 &= \mathbf{0}, \end{aligned} \tag{C.26}$$

so

$$\mathbf{f}_{\text{eq}} = \begin{pmatrix} \pi_0 \\ 1-\pi_0 \\ 0 \\ 0 \end{pmatrix}. \tag{C.27}$$

When $\alpha_1 > \alpha_0$,

$$\overline{X}_{\text{eq}} = \alpha_1, \tag{C.28}$$

from which it follows that

$$\begin{aligned} \mathbf{f}_0 &= \mathbf{0} \\ \mathbf{f}_1 &\propto \mathbf{p}_1, \end{aligned} \tag{C.29}$$

bioRxiv preprint doi: https://doi.org/10.64898/2026.03.15.711953; this version posted March 16, 2026. The copyright holder for this preprint (which was not certified by peer review) is the author/funder, who has granted bioRxiv a license to display the preprint in perpetuity. It is made available under aCC-BY 4.0 International license.

so

$$\mathbf{f}_{\text{eq}} = \begin{pmatrix} 0 \\ 0 \\ \pi_1 \\ 1-\pi_1 \end{pmatrix}. \tag{C.30}$$

When $\alpha_0 = \alpha_1$, the principal eigenvector is degenerate and any linear combination of the above two eigenvectors will be a valid equilibrium eigenvector.

Limiting case: zero fitness of phenotype 1 ($X^{(0)} = 0$). We now explore the limiting case where $X^{(1)} = 0$, some simplifications can be made. In this limit,

$$\alpha_g = \pi_g X^{(0)}, \tag{C.31}$$

from which it follows that

$$\overline{X}_{\text{eq}} = X^{(0)}\lambda, \tag{C.32}$$

where we define

$$\lambda = \frac{(1-\mu)(\pi_0+\pi_1) + \sqrt{(1-\mu)^2(\pi_0+\pi_1)^2 - 4\pi_0\pi_1(1-2\mu)}}{2}. \tag{C.33}$$

now, we note that the fraction

$$\frac{\alpha_0(2\mu-1)}{\overline{X}_{\text{eq}}} = \frac{X^{(0)}\pi_0(2\mu-1)}{X^{(0)}\lambda} = \frac{\pi_0(2\mu-1)}{\lambda} \tag{C.34}$$

loses dependence on the the fitness of phenotype 0. As a result, the equilibrium frequency vector no longer has any dependence on fitness whatsoever:

$$\mathbf{f}_{\text{eq}} = \frac{1}{1+\frac{\pi_0(2\mu-1)}{\lambda}} \begin{pmatrix} \mu\pi_0 \\ \mu(1-\pi_0) \\ \left[(1-\mu)+\frac{\pi_0(2\mu-1)}{\lambda}\right]\pi_1 \\ \left[(1-\mu)+\frac{\pi_0(2\mu-1)}{\lambda}\right](1-\pi_1) \end{pmatrix}. \tag{C.35}$$

C.3 Per-genotype effective fitness and phenotypic buoying

The counterintuitive "surprise" that the genotype 1, phenotype 1 (red node in Main Text) beats genotype 0, phenotype 0 (blue node) despite having lower mapping probability and fitness is actually well-justified by ProP Gen theory. Recall that classical selection dynamics with phenotype noise during replication in the ProP Gen equations can be condensed into a collective term "PrGP selection":

$$\begin{aligned} \frac{\partial f_g^{(p)}}{\partial t} &= \underbrace{f_g^{(p)}(t)(X^{(p)} - \overline{X}(t))}_{\text{classical selection}} + \underbrace{\sum_{k\neq p}\left[X^{(k)} f_g^{(k)}(t)\varphi_g^{(k\to p)} - X^{(p)} f_g^{(p)}(t)\varphi_g^{(p\to k)}\right]}_{\text{phenotype noise at birth}} \\ &= \underbrace{\sum_{k\in P} f_g^{(k)}(t)\varphi_g^{(k\to p)} X^{(k)} - f_g^{(p)}(t)\overline{X}(t)}_{\text{"PrGP selection"}}. \end{aligned} \tag{C.36}$$

Summing over all phenotypes p, we can write a differential equation for the genotype frequencies:

$$\frac{\partial f_g}{\partial t} = f_g(t)\left(\frac{\sum_{k\in P} f_g^{(k)}(t)X^{(k)}}{\sum_{k\in P} f_g^{(k)}(t)} - \overline{X}(t)\right), \tag{C.37}$$

which simply looks like classical selection with a frequency and time-dependent per-genotype "effective fitness" given by the ratio within the parentheses above

$$X_g^{\text{eff}}(t) \equiv \frac{\sum_{k\in P} f_g^{(k)}(t)X^{(k)}}{\sum_{k\in P} f_g^{(k)}(t)}. \tag{C.38}$$

bioRxiv preprint doi: https://doi.org/10.64898/2026.03.15.711953; this version posted March 16, 2026. The copyright holder for this preprint (which was not certified by peer review) is the author/funder, who has granted bioRxiv a license to display the preprint in perpetuity. It is made available under aCC-BY 4.0 International license.

This shows that, for each genotype, PrGP selection makes a genotype-only fitness landscape appear *time-dependent*, and the genotype's growth rate is exactly the per-genotype average fitness. Thus, a positive feedback loop ensues where genotype 1, phenotype 0 (green node), which is high fitness and high probability, helps genotype 1 overall absorb more population density, which of course accelerates the acquisition of more population density. As time goes by, the effective fitness of genotype 1 increases rapidly, and the red node benefits from the green node shunting its population density to the red node via phenotype noise.

C.4 Exact phase diagrams for equilibrium frequency distributions for phenotypic buoys with 2 genotypes and 2 phenotypes

In the DGP classical limit, suppose there are two alleles. In the infinite population limit (no genetic drift) with no mutations, where only selection dominates the dynamics, the allele with higher fitness will approach frequency 1 as the system approaches equilibrium, while the allele with lower fitness will approach frequency 0. With mutations, it is possible for both alleles to coexist at equilibrium. With PrGP maps, we showed analytically that, in general, multiple phenotypes can coexist at equilibrium, even when there are no mutations. We calculated the equilibrium frequencies of GP pairs in the two genotype, two phenotype scenario in eq. (C.23).

Here, we exactly calculate theoretical phase diagrams of coexistence between the four possible GP pairs in the two genotype, two phenotype scenario. The "phases" are relative orderings (permutations) of the equilibrium frequencies of the four GP pairs, assuming that no two frequencies are perfectly equal. There are $4! = 24$ possible orderings. To more conveniently explore the phase diagram in a two-dimensional visualization, we select the probability of genotype 1 mapping to phenotype 0 ($\pi_1 = \varphi_1^{(0)}$) and the mutation rate (μ) to be the controllable variables in the phase diagram. We fix the phenotype fitnesess $X^{(0)} = 0.09$ and $X^{(1)} = 0.02$. We note that, since $f_{0,\text{eq}}^{(0)} = \mu\pi_0$ and $f_{0,\text{eq}}^{(1)} = (1-\mu)\pi_0$, fixing π_0 guarantees an equilibrium ordering of $f_{0,\text{eq}}^{(0)}$ and $f_{0,\text{eq}}^{(1)}$. We will also fix π_0 to three different values ($\pi_0 = 0.4 < 0.5$, $\pi_0 = 0.5$, and $\pi_0 = 0.6 > 0.5$) in order to explore the shape of the (π_1, μ) phase diagram in different π_0 regimes. This reduces the maximum possible number of phases visible on the (π_1, μ) phase diagram to $4!/2 = 12$, though the real number may be less than 12 based on other constraints.

The phase boundaries can be computed by finding the conditions when any two of the equilibrium frequencies are equal. There are 4 frequencies, so that means there are $\binom{4}{2} = 6$ equations to consider. We handle each case below, ignoring the normalization constant common to all terms:

1. $f_{0,\text{eq}}^{(0)} = f_{0,\text{eq}}^{(1)}$, which is

$$\mu\pi_0 = \mu(1-\pi_0), \tag{C.39}$$

which simplifies to

$$\pi_0 = 0.5. \tag{C.40}$$

This curve will thus not be found on the (π_1, μ), but we can expect that the shape of the (π_1, μ) may change depending on the value of π_0, which we will illustrate soon.

2. $f_{1,\text{eq}}^{(0)} = f_{1,\text{eq}}^{(1)}$, which is

$$\left[(1-\mu) + \frac{\alpha_0(2\mu-1)}{\overline{X}_{\text{eq}}}\right]\pi_1 = \left[(1-\mu) + \frac{\alpha_0(2\mu-1)}{\overline{X}_{\text{eq}}}\right](1-\pi_1). \tag{C.41}$$

which simplifies to

$$\pi_1 = 0.5. \tag{C.42}$$

This curve will appear as a vertical line in the (π_1, μ) phase diagram.

3. $f_{0,\text{eq}}^{(0)} = f_{1,\text{eq}}^{(1)}$, which is

$$\mu\pi_0 = \left[(1-\mu) + \frac{\alpha_0(2\mu-1)}{\overline{X}_{\text{eq}}}\right](1-\pi_1). \tag{C.43}$$

This will be an implicitly defined curve in the (π_1, μ) space which can be solved numerically for plotting.

bioRxiv preprint doi: https://doi.org/10.64898/2026.03.15.711953; this version posted March 16, 2026. The copyright holder for this preprint (which was not certified by peer review) is the author/funder, who has granted bioRxiv a license to display the preprint in perpetuity. It is made available under aCC-BY 4.0 International license.

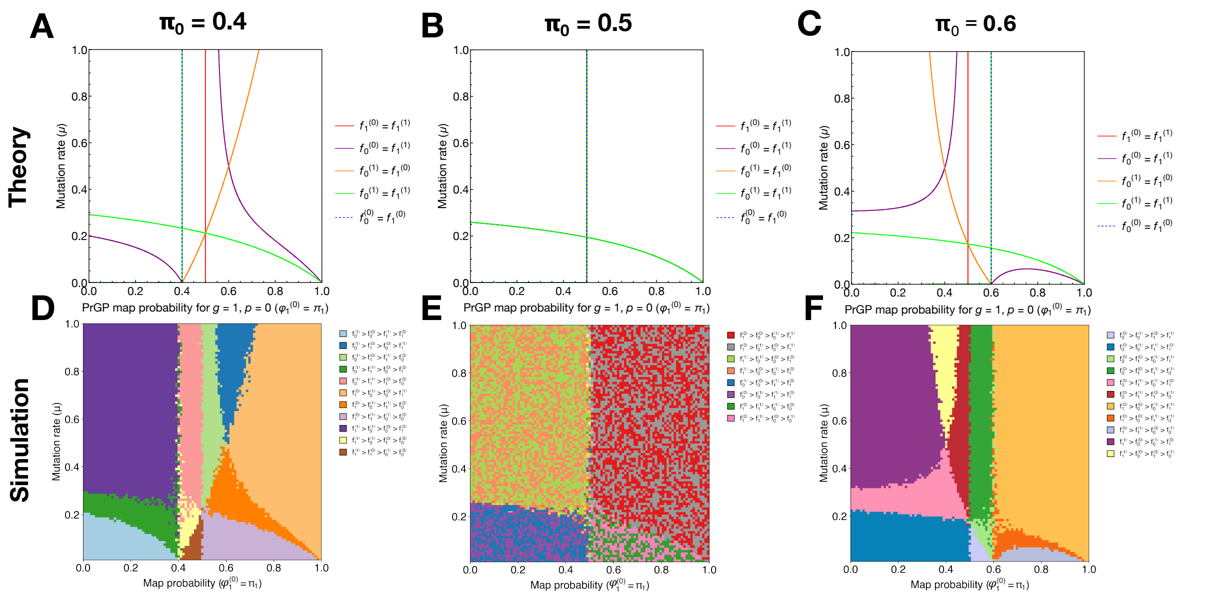

Figure S1: **Complex phase diagrams of coexistence between genotype-phenotype pairs at equilibrium.** (**A-C**) Theoretical phase boundaries for genotype-phenotype pair orderings at equilibrium for $\pi_0 = \{0.4, 0.5, 0.6\}$. (**D-F**) Corresponding Numerical phase diagrams from `ProSeD` for $\pi_0 = \{0.4, 0.5, 0.6\}$.

4. $f^{(1)}_{0,\text{eq}} = f^{(0)}_{1,\text{eq}}$, which is

$$\mu(1-\pi_0) = \left[(1-\mu) + \frac{\alpha_0(2\mu-1)}{\overline{X}_{\text{eq}}}\right]\pi_1. \tag{C.44}$$

This will be an implicitly defined curve in the (π_1, μ) space which can be solved numerically for plotting.

5. $f^{(1)}_{0,\text{eq}} = f^{(1)}_{1,\text{eq}}$, which is

$$\mu(1-\pi_0) = \left[(1-\mu) + \frac{\alpha_0(2\mu-1)}{\overline{X}_{\text{eq}}}\right](1-\pi_1). \tag{C.45}$$

This will be an implicitly defined curve in the (π_1, μ) space which can be solved numerically for plotting.

6. $f^{(0)}_{0,\text{eq}} = f^{(0)}_{1,\text{eq}}$, which is

$$\mu\pi_0 = \left[(1-\mu) + \frac{\alpha_0(2\mu-1)}{\overline{X}_{\text{eq}}}\right]\pi_1. \tag{C.46}$$

This will be an implicitly defined curve in the (π_1, μ) space which can be solved numerically for plotting.

D Phenotypic bridges

We now develop the theory of *phenotypic bridges*. Fitness valleys in the presence of DGP mapping require either operating in sufficiently polymorphic regime such that the population can spread out over multiple genotypes and eventually discover a distant peak, or it requires stochastic tunneling. Thus, valley crossing can be a very slow process especially when mutation rates are low or when the valley is very deep. To analytically investigate valley crossing in the presence of a "phenotypic bridge," we consider three genotypes: 0, 1, and 2, and two phenotypes: 0 and 1. We assume that genotypes 0 and 2 deterministically map onto phenotype 0 (i.e. with probability 1), and that genotype 1 either maps onto phenotype 0 with probability π or onto phenotype 1 with probability $1-\pi$. We also assume that phenotype mapping probabilities do not depend on the initial phenotype, so $\varphi_g^{(k\to p)} = \varphi_g^{(p)}$. Thus, we have $\varphi_0^{(0)} = 1$, $\varphi_0^{(1)} = 0$, $\varphi_1^{(0)} = \pi$, $\varphi_1^{(1)} = 1-\pi$, $\varphi_2^{(0)} = 1$, and $\varphi_2^{(1)} = 0$. We also define shorthand

bioRxiv preprint doi: https://doi.org/10.64898/2026.03.15.711953; this version posted March 16, 2026. The copyright holder for this preprint (which was not certified by peer review) is the author/funder, who has granted bioRxiv a license to display the preprint in perpetuity. It is made available under aCC-BY 4.0 International license.

labels for each of the 4 relevant genotype-phenotype pairs: the starting node has frequency $f_s \equiv f_0^{(0)}$, the bridge node has frequency $f_b \equiv f_1^{(0)}$, the valley node has frequency $f_v \equiv f_1^{(1)}$, and the ending node has frequency $f_e \equiv f_2^{(0)}$. The mutation rates are determined by first selecting offspring to mutate with probability μ, and then a mutational neighbor is chosen with uniform probability, leading to a mutation matrix elements $\mu_{0\to1} = \mu_{2\to1} = \mu$ and $\mu_{1\to0} = \mu_{1\to2} = \mu/2$. We assign fitnesses $X^{(0)} = X_0$ and $X^{(1)} = \gamma X_0$, with $0 \leq \gamma < 1$. Now, we work in the diffusion limit with infinite population limit, assuming mutation probability at birth to be μ, and additionally only considering phenotype noise at birth:

$$\frac{\partial f_g^{(p)}}{\partial t} = \underbrace{\varphi_g^{(p)} \sum_{k\in P} f_g^{(k)} X^{(k)} - f_g^{(p)}(t)\overline{X}(t)}_{\text{PrGP selection}} + \underbrace{\varphi_g^{(p)} \sum_{h\neq g} \sum_{k\in P} X^{(k)} \left[f_h^{(k)} \mu_{h\to g} - f_g^{(k)}(t)\mu_{g\to h} \right]}_{\text{mutation upon replication}}. \tag{D.1}$$

For each of the four genotype-phenotype pairs, we can write the differential equations

$$\begin{aligned}
\frac{\partial f_s}{\partial t} &= f_s(X_0 - \overline{X}(t)) + \frac{\mu}{2} X_0 f_b + \frac{\mu}{2} X_0 \gamma f_v - \mu X_0 f_s \\
\frac{\partial f_e}{\partial t} &= f_e(X_0 - \overline{X}(t)) + \frac{\mu}{2} X_0 f_b + \frac{\mu}{2} X_0 \gamma f_v - \mu X_0 f_e \\
\frac{\partial f_b}{\partial t} &= \pi(f_b X_0 + f_v X_0 \gamma) - f_b \overline{X}(t) + \pi\mu X_0(f_s + f_e) - \pi\mu(f_b X_0 + f_v X_0 \gamma) \\
\frac{\partial f_v}{\partial t} &= (1-\pi)(f_b X_0 + f_v X_0 \gamma) - f_v \overline{X}(t) + (1-\pi)\mu X_0(f_s + f_e) - (1-\pi)\mu(f_b X_0 + f_v X_0 \gamma).
\end{aligned} \tag{D.2}$$

D.1 Exact equilibrium distribution

At equilibrium, the time derivatives vanish, and we can take the mean fitness-dependent terms to the left-hand side of the equation, setting up an eigenvalue equation for the equilibrium frequency vectors:

$$\begin{aligned}
\overline{X}^{\text{eq}} f_s^{\text{eq}} &= f_s^{\text{eq}} X_0 + \frac{\mu}{2} X_0 f_b^{\text{eq}} + \frac{\mu}{2} X_0 \gamma f_v^{\text{eq}} - \mu X_0 f_s^{\text{eq}} \\
\overline{X}^{\text{eq}} f_e^{\text{eq}} &= f_e^{\text{eq}} X_0 + \frac{\mu}{2} X_0 f_b^{\text{eq}} + \frac{\mu}{2} X_0 \gamma f_v^{\text{eq}} - \mu X_0 f_e^{\text{eq}} \\
\overline{X}^{\text{eq}} f_b^{\text{eq}} &= \pi(f_b^{\text{eq}} X_0 + f_v^{\text{eq}} X_0 \gamma) + \pi\mu X_0(f_s^{\text{eq}} + f_e^{\text{eq}}) - \pi\mu(f_b^{\text{eq}} X_0 + f_v^{\text{eq}} X_0 \gamma) \\
\overline{X}^{\text{eq}} f_v^{\text{eq}} &= (1-\pi)(f_b^{\text{eq}} X_0 + f_v^{\text{eq}} X_0 \gamma) + (1-\pi)\mu X_0(f_s^{\text{eq}} + f_e^{\text{eq}}) - (1-\pi)\mu(f_b^{\text{eq}} X_0 + f_v^{\text{eq}} X_0 \gamma).
\end{aligned} \tag{D.3}$$

Diving the third and fourth equations, we immediately find

$$\frac{f_b^{\text{eq}}}{f_v^{\text{eq}}} = \frac{\pi}{1-\pi}. \tag{D.4}$$

From the first two equations (and by symmetry of the setup), we can also see

$$f_s^{\text{eq}} = f_e^{\text{eq}}. \tag{D.5}$$

We also note that all equations can be divided by X_0, to yield equations independent of X_0, since

$$\overline{X}^{\text{eq}} = X_0(f_s^{\text{eq}} + f_e^{\text{eq}} + f_b^{\text{eq}} + \gamma f_v^{\text{eq}}). \tag{D.6}$$

Defining

$$\overline{x}^{\text{eq}} \equiv \frac{\overline{X}}{X_0} = f_s^{\text{eq}} + f_e^{\text{eq}} + f_b^{\text{eq}} + \gamma f_v^{\text{eq}}, \tag{D.7}$$

and

$$w \equiv f_b^{\text{eq}} + \gamma f_v^{\text{eq}} \tag{D.8}$$

$$v \equiv f_s^{\text{eq}} + f_e^{\text{eq}} = 2 f_s^{\text{eq}} = 2 f_e^{\text{eq}} \tag{D.9}$$

and making additional rearrangements, we now have

$$\begin{aligned}
\overline{x}^{\text{eq}} f_s^{\text{eq}} &= f_s(1-\mu) + \frac{\mu w}{2} \\
\overline{x}^{\text{eq}} f_e^{\text{eq}} &= f_e(1-\mu) + \frac{\mu w}{2} \\
\overline{x}^{\text{eq}} f_b^{\text{eq}} &= \pi w + \pi\mu v - \pi\mu w \\
\overline{x}^{\text{eq}} f_v^{\text{eq}} &= (1-\pi) w + (1-\pi)\mu v - (1-\pi)\mu w.
\end{aligned} \tag{D.10}$$

bioRxiv preprint doi: https://doi.org/10.64898/2026.03.15.711953; this version posted March 16, 2026. The copyright holder for this preprint (which was not certified by peer review) is the author/funder, who has granted bioRxiv a license to display the preprint in perpetuity. It is made available under aCC-BY 4.0 International license.

Adding the first two equations, we have

$$\overline{x}^{\text{eq}} v = v(1-\mu) + \mu w. \tag{D.11}$$

Then adding the third equation to the fourth equation multiplied by γ, we have

$$\overline{x}^{\text{eq}} w = \theta[w(1-\mu) + \mu v], \tag{D.12}$$

where we have defined

$$\theta \equiv \pi + \gamma(1-\pi). \tag{D.13}$$

We can now write eq. (D.11) and eq. (D.12) as a 2×2 matrix equation

$$\overline{x}^{\text{eq}} \begin{pmatrix} v \\ w \end{pmatrix} = \begin{pmatrix} 1-\mu & \mu \\ \theta\mu & \theta(1-\mu) \end{pmatrix} \begin{pmatrix} v \\ w \end{pmatrix}. \tag{D.14}$$

The characteristic equation is

$$[1-\mu-\overline{x}^{\text{eq}}][\theta(1-\mu) - \overline{x}^{\text{eq}}] - \theta\mu^2 = 0. \tag{D.15}$$

Solving the quadratic equation for $\overline{x}^{\text{eq}}$ and taking the positive solution, we have

$$\overline{x}^{\text{eq}} = \frac{(1+\theta)(1-\mu) + \sqrt{(1+\theta)^2(1-\mu)^2 - 4\theta(1-2\mu)}}{2}. \tag{D.16}$$

We can now rearrange eq. (D.11) to write

$$\frac{v}{w} = \frac{\mu}{\overline{x}^{\text{eq}} - (1-\mu)} \tag{D.17}$$

From eq. (D.4), we can write

$$f_b^{\text{eq}} = \pi(f_b^{\text{eq}} + f_v^{\text{eq}}) \tag{D.18}$$

and

$$f_v^{\text{eq}} = (1-\pi)(f_b^{\text{eq}} + f_v^{\text{eq}}). \tag{D.19}$$

Now, it follows that

$$w = f_b^{\text{eq}} + \gamma f_v^{\text{eq}} = (f_b^{\text{eq}} + f_v^{\text{eq}})(\pi + \gamma(1-\pi)) = (f_b^{\text{eq}} + f_v^{\text{eq}})\theta. \tag{D.20}$$

Thus, we can write

$$f_b^{\text{eq}} + f_v^{\text{eq}} = \frac{w}{\theta}. \tag{D.21}$$

Now, we can use normalization of all four genotype-phenotype pair frequencies to write

$$\begin{aligned} 1 &= f_s^{\text{eq}} + f_e^{\text{eq}} + f_b^{\text{eq}} + f_v^{\text{eq}} \\ &= v + \frac{w}{\theta} \\ &= \frac{\mu}{\overline{x}^{\text{eq}} - (1-\mu)} w + \frac{w}{\theta}. \end{aligned} \tag{D.22}$$

Solving for w, we have

$$w = \frac{\theta}{\theta\left(\frac{\mu}{\overline{x}^{\text{eq}} - (1-\mu)}\right) + 1} = \frac{\theta(\overline{x}^{\text{eq}} - (1-\mu))}{\theta\mu + \overline{x}^{\text{eq}} - (1-\mu)}. \tag{D.23}$$

We can also write v:

$$v = \frac{\theta(\overline{x}^{\text{eq}} - (1-\mu))}{\theta\mu + \overline{x}^{\text{eq}} - (1-\mu)} \frac{\mu}{\overline{x}^{\text{eq}} - (1-\mu)} = \frac{\theta\mu}{\theta\mu + \overline{x}^{\text{eq}} - (1-\mu)}. \tag{D.24}$$

We can now write the equilibrium frequency vector, using $v = 2f_s^{\text{eq}} = 2f_e^{\text{eq}}$ and $w = \theta f_b^{\text{eq}}/\pi = \theta f_v^{\text{eq}}/(1-\pi)$:

$$\mathbf{f}^{\text{eq}} = \begin{pmatrix} f_s^{\text{eq}} \\ f_e^{\text{eq}} \\ f_b^{\text{eq}} \\ f_v^{\text{eq}} \end{pmatrix} = \frac{1}{\theta\mu + \overline{x}^{\text{eq}} - (1-\mu)} \begin{pmatrix} \frac{\theta\mu}{2} \\ \frac{\theta\mu}{2} \\ \pi([\overline{x}^{\text{eq}} - (1-\mu)] \\ (1-\pi)[\overline{x}^{\text{eq}} - (1-\mu)] \end{pmatrix}, \tag{D.25}$$

where $\overline{x}^{\text{eq}}$ is given by eq. (D.16).

bioRxiv preprint doi: https://doi.org/10.64898/2026.03.15.711953; this version posted March 16, 2026. The copyright holder for this preprint (which was not certified by peer review) is the author/funder, who has granted bioRxiv a license to display the preprint in perpetuity. It is made available under aCC-BY 4.0 International license.

D.2 Relaxation time constant

We now linearize the nonlinear diffusion equations in eq. (D.2) around the equilibrium frequency vector and compute the time constant for the slowest decay mode toward equilibrium. Expanding the nonlinear equation around the equilibrium vector $\mathbf{f} = \mathbf{f}^{\mathrm{eq}}$, we obtain

$$\frac{\partial \mathbf{f}}{\partial t} = J\mathbf{f}, \tag{D.26}$$

where J is the Jacobian

$$J = \begin{pmatrix} 1 - \overline{x}^{\mathrm{eq}} - f_s^{\mathrm{eq}} - \mu & -f_s^{\mathrm{eq}} & -f_s^{\mathrm{eq}} + \frac{\mu}{2} & -f_s^{\mathrm{eq}}\gamma + \frac{\mu\gamma}{2} \\ -f_s^{\mathrm{eq}} & 1 - \overline{x}^{\mathrm{eq}} - f_s^{\mathrm{eq}} - \mu & -f_s^{\mathrm{eq}} + \frac{\mu}{2} & -f_s^{\mathrm{eq}}\gamma + \frac{\mu\gamma}{2} \\ -f_b^{\mathrm{eq}} + \pi\mu & -f_b^{\mathrm{eq}} + \pi\mu & \pi(1-\mu) - \overline{x}^{\mathrm{eq}} - f_b^{\mathrm{eq}} & \pi(1-\mu)\gamma - f_b^{\mathrm{eq}}\gamma \\ -f_v^{\mathrm{eq}} + (1-\pi)\mu & -f_v^{\mathrm{eq}} + (1-\pi)\mu & (1-\pi)(1-\mu) - f_v^{\mathrm{eq}} & (1-\pi)(1-\mu)\gamma - \overline{x}^{\mathrm{eq}} - f_v^{\mathrm{eq}}\gamma \end{pmatrix}. \tag{D.27}$$

Due to normalization, one of the eigenvalues will be 0. Numerical evidence from the main text makes it clear that the bridge and valley genotype-phenotype pairs rapidly equilibriate while the slowest decay mode is the relaxation of f_s and f_e, which appear to "mirror" each other while decaying toward exponential—that is, f_s and f_e experience equal but opposite changes while relaxing toward equilibrium. By inspection, it is readily verifiable from the Jacobian above that the vector

$$\mathbf{f}^{\mathrm{slow}} = \begin{pmatrix} -1 \\ 1 \\ 0 \\ 0 \end{pmatrix} \tag{D.28}$$

is indeed an eigenvector of the Jacobian. The corresponding eigenvalue,

$$\lambda^{\mathrm{slow}} = (1-\mu) - \overline{x}^{\mathrm{eq}} \tag{D.29}$$

provides the exponential decay rate of the slowest mode. The associated exponential time constant for equilibrating over the bridge is

$$\begin{aligned} \tau &= -\frac{1}{\lambda^{\mathrm{slow}}} \\ &= \frac{1}{\overline{x}^{\mathrm{eq}} - (1-\mu)} \\ &= \frac{2}{\sqrt{(1+\theta)^2(1-\mu)^2 - 4\theta(1-2\mu)} - (1-\theta)(1-\mu)} \\ &= \frac{2}{\sqrt{(1+\pi+\gamma(1-\pi))^2(1-\mu)^2 - 4(\pi+\gamma(1-\pi))(1-2\mu)} - (1-\pi+\gamma(1-\pi))(1-\mu)} \end{aligned} \tag{D.30}$$

where we used $\theta = \pi + \gamma(1-\pi)$, as defined earlier. We show in the main text that this theoretical time constant displays excellent agreement with numerical fits from exponentially fitting trajectory data.

E Selection dynamics: global translational symmetry breaking of the fitness landscape

For the remainder of this supplementary material, we assume that the phenotype probability does not depend on the starting phenotype, so $\varphi_g^{(k\to p)} \equiv \varphi_g^{(p)}$, but rather only depends on the destination phenotype.

E.1 Global translational symmetry breaking of the fitness landscape

We now consider the selection term in the DGP and PrGP cases and show that the uncertain GP mapping in the latter case leads to dependence of the evolutionary dynamics on absolute, not simply, relative fitnesses. This also occurs when comparing the Eigen quasispecies equations with Kimura's diffusion

bioRxiv preprint doi: https://doi.org/10.64898/2026.03.15.711953; this version posted March 16, 2026. The copyright holder for this preprint (which was not certified by peer review) is the author/funder, who has granted bioRxiv a license to display the preprint in perpetuity. It is made available under aCC-BY 4.0 International license.

limit with mutations; the former couples mutations to fitnesses, while the latter treats mutations as an additive generator. Here, phenotype noise during replication is coupled to fitness while stochastic phenotype switching is an additive contribution.

The DGP selection term (i.e. the replicator equation) is:

$$\frac{\partial f_g}{\partial t} = f_g(t)(X_g - \overline{X}(t)), \tag{E.1}$$

and the PrGP selection term (which we showed previously is the same as the DGP selection term plus a fitness-dependent phenotype switching term) has dependence on the GP mapping probability $\varphi_g^{(p)}$:

$$\frac{\partial f_g^{(p)}}{\partial t} = \varphi_g^{(p)} \sum_{k\in P} f_g^{(k)}(t) X^{(k)} - f_g^{(p)}(t)\overline{X}(t). \tag{E.2}$$

We now consider a shift of the entire fitness landscape by some absolute fitness A, so that all fitnesses are translated additively:

$$X_g \mapsto X_g + A \quad \text{or} \quad X^{(p)} \mapsto X^{(p)} + A. \tag{E.3}$$

The DGP selection term becomes:

$$\begin{aligned}
\frac{\partial f_g}{\partial t} &= f_g(t)\left[(X_g + A) - \sum_{g\in G} f_g(t)(X_g + A)\right], \\
&= f_g(t)\left[(X_g + A) - \sum_{g\in G} f_g(t)X_g + A\sum_{g\in G} f_g(t)\right] \\
&= f_g(t)\left[(X_g + A) - \sum_{g\in G} f_g(t)X_g + A\right] \\
&= f_g(t)(X_g - \overline{X}(t)),
\end{aligned} \tag{E.4}$$

which means it does not transform at all. Akin to how only relative differences in potential energy impact trajectories in classical mechanics, only relative differences in growth rates affect evolutionary dynamics in the deterministic GP mapping case, as is well known. The PrGP selection term, however, demonstrates a major difference:

$$\begin{aligned}
\frac{\partial f_g^{(p)}}{\partial t} &= \varphi_g^{(p)} \sum_{k\in P} f_g^{(k)}(t)(X^{(k)} + A) - f_g^{(p)}(t) \sum_{g\in G}\sum_{k\in P} f_g^{(k)}(t)(X^{(k)} + A) \\
&= \varphi_g^{(p)} \sum_{k\in P} f_g^{(k)}(t)X^{(k)} + A\varphi_g^{(p)} \sum_{k\in P} f_g^{(k)}(t) \\
&\quad - f_g^{(p)}(t) \sum_{g\in G}\sum_{k\in P} f_g^{(p)}(t)X^{(p)} - Af_g^{(p)}(t)\sum_{g\in G}\sum_{k\in P} f_g^{(k)}(t) \\
&= \varphi_g^{(p)} \sum_{k\in P} f_g^{(k)}(t)X^{(k)} - f_g^{(p)}(t)\overline{X}(t) + A\left[\varphi_g^{(p)}\sum_{k\in P} f_g^{(k)}(t) - f_g^{(p)}(t)\right].
\end{aligned} \tag{E.5}$$

Thus, in the PrGP case there remains a dependence on A, indicating that *absolute* fitness, and not simply relative fitness (as is the case in canonical population genetics), determines evolutionary dynamics.

E.2 Mean fitness can decrease with time.

Moreover, we now construct an example to show that it is possible for the change in mean fitness to be *negative*. We are searching for a class of solutions where

$$\sum_{g\in G}\left(\sum_{p\in P} X^{(p)}\varphi_g^{(p)}\right)\left(\sum_{k\in P} X^{(k)} f_g^{(k)}\right) - \overline{X}(t)^2 < 0. \tag{E.6}$$

bioRxiv preprint doi: https://doi.org/10.64898/2026.03.15.711953; this version posted March 16, 2026. The copyright holder for this preprint (which was not certified by peer review) is the author/funder, who has granted bioRxiv a license to display the preprint in perpetuity. It is made available under aCC-BY 4.0 International license.

Consider a system with two genotypes and two phenotypes, each marked with indices 0 and 1. Suppose we have fitnesses

$$X^{(0)} = 0.09 \quad \text{and} \quad X^{(1)} = 0.02, \tag{E.7}$$

GP mapping probabilities

$$\varphi_0^{(0)} = 0.1, \quad \varphi_0^{(1)} = 0.9, \quad \varphi_1^{(0)} = 0.8, \quad \text{and} \quad \varphi_1^{(1)} = 0.2, \tag{E.8}$$

and initial frequencies

$$f_0^{(0)} = 0.4, \quad f_0^{(1)} = 0.1, \quad f_1^{(0)} = 0.05, \quad \text{and} \quad f_1^{(1)} = 0.45. \tag{E.9}$$

The mean fitness is

$$\overline{X}(0) = \sum_{g\in G}\sum_{p\in P} X^{(p)} f_g^{(p)}(0) = (0.0515)^2 = 0.00265225. \tag{E.10}$$

The first term in the expression eq. (E.6) is

$$\sum_{g\in G}\left(\sum_{p\in P} X^{(p)}\varphi_g^{(p)}\right)\left(\sum_{k\in P} X^{(k)} f_g^{(k)}\right) = 0.002052. \tag{E.11}$$

Since $0.002052 < 0.005776$, we have successfully found an example where

$$\frac{\mathrm{d}\overline{X}}{\mathrm{d}t} = -0.00060025 < 0, \tag{E.12}$$

which means that mean fitness can decrease *only with selection* dynamics. We simulate this particular case with the initial conditions given above in Figure S2C-E. A summary of the probabilities, fitnesses, and genotype network topology is shown in Figure S2C. Frequency trajectories can show non-monotonicity (Figure S2D). Genotype 0, phenotype 1 (shown in orange), has high GP mapping probability but is low fitness. Thus, asymptotically, its frequency will be low. But, initially, its frequency grows because of the relatively high initial frequency of genotype 0, phenotype 0 (blue). The rapid growth of the genotype 0, phenotype 1 and non-monotonic behavior contributes to the initial decrease in mean fitness before its eventual increase towards its equilibrium value (Figure S2E).

Over the last 90 years, many have argued against the validity Fisher's fundamental theorem of natural selection, suggesting that misinterpretation of the theorem, mutations, environmental interactions, epistasis, linkage disequilibrium, and other factors can prevent mean fitness from increasing with time. We have now shown that probabilistic mapping of genotype to phenotype can also cause decrease in mean fitness. Unlike classical DGP selection, where genotype frequencies tend towards fixation if they have the uniquely highest fitness, in PrGP selection, high fitness phenotypes can still "feed" lower fitness phenotypes. We discuss this phenomenon, which we call "phenotype buoys," in depth in the following section.

F Exact persister cell resuscitation dynamics

Here, we construct ProP Gen theory equations for phenotype noise and SPS associated with phenotypic resuscitation dynamics. We then show that they are exactly solvable. In the experiments of Fang and Allison[36], they allow persister cells to resuscitate after washing away antibiotics. The dormant persister cells (P) can then resuscitate into three active phenotypes: failed (F), damaged (D), and healthy (H). Dormant persisters only resuscitate but they do not replicate until they have transitioned to an active phenotype, so we set the dormant fitness to zero $X^{(P)}$. The damaged and failed phenotypes both are morphologically aberrant, but the failed ones cannot replicate, so we set their fitness to zero $X^{(F)} = 0$. Damaged active cells can still replicate with fitness $X^{(D)}$ which is presumed to be less than or equal to the healthy cells' fitness $X^{(H)}$.

The transition rate (which is an SPS rate $S(t)$) out of the dormant persister state is empirically measured to be exponential in time[36], which is highly suggestive of a positive feedback mechanism. They empirically validate the form:

$$S(t) = \alpha e^{\beta t}. \tag{F.1}$$

bioRxiv preprint doi: https://doi.org/10.64898/2026.03.15.711953; this version posted March 16, 2026. The copyright holder for this preprint (which was not certified by peer review) is the author/funder, who has granted bioRxiv a license to display the preprint in perpetuity. It is made available under aCC-BY 4.0 International license.

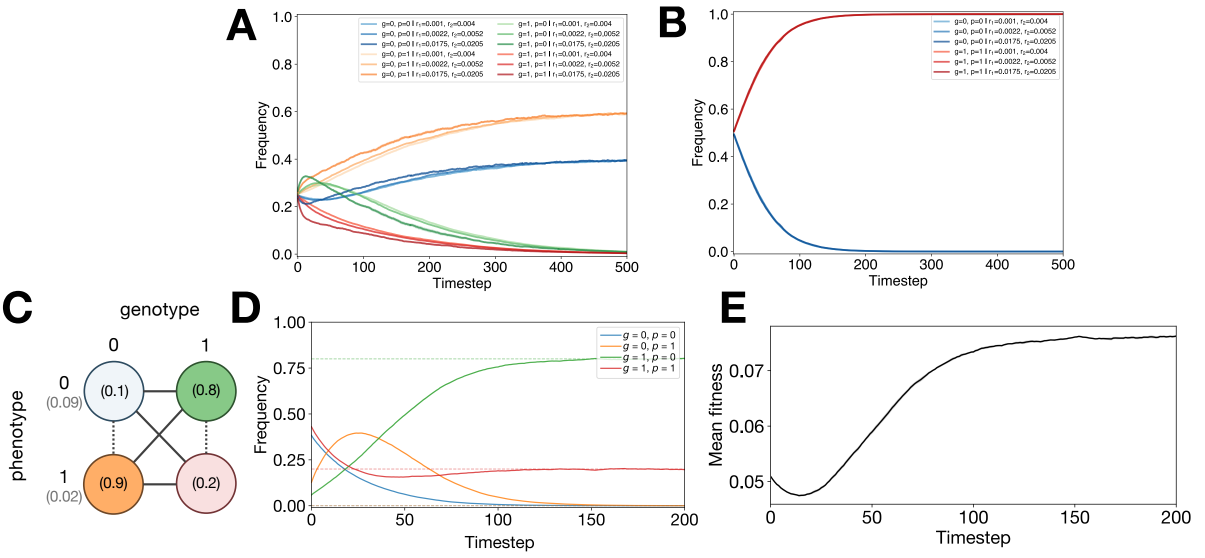

Figure S2: **Phenotypic uncertainty creates a dependence on absolute fitness.** (**A**) PrGP dynamics translated by different absolute fitnesses while keeping relative fitnesses the same. Translational symmetry breaking is observed. (**B**) DGP dynamics at different absolute fitnesses. Translational invariance is observed. (**C**) Diagram of two genotype, two phenotype set up for the PrGP cases. (**D**) `ProSeD` simulation of the system with the specified initial frequencies from Section C.2. (**E**) Mean fitness versus time.

Thus, the number of persister cells decays as an exponential of an exponential. We call the SPS probabilities out of the dormant state $\sigma^{(P\to i)}$ for $i \in \{D, F, H\}$, and $\sum_i \sigma^{(P\to i)} = 1$. Furthermore, the damaged state's cell division ("partitioning") can be with compromised fidelity, either creating more damaged cells or creating healthy cells. This is a key example of phenotype noise at birth, in contrast to SPS. Thus, we have $\varphi^{D\to H}$ as the probability that the offspring of a damaged cell is healthy, and $\varphi^{D\to D} = 1 - \varphi^{D\to H}$ is the probability that the offspring of a damaged cell is damaged.

Given the phenotypic transitions described above, we can now write down the ProP Gen equations for the four phenotypes. However, it is also important to note that Fang and Allison [36] did not impose any population bottleneck (i.e. via serial dilution), so if we were to redo the derivation from Section A, we would not have a mean fitness term imposing selection due to Malthusian fitnesses. We also will not include the effect of sampling noise via genetic drift for the same reason. Further fluctuations would be due to noise in the parameters themselves such as fitnesses and the mapping or SPS probabilities. Thus, we write down the ODEs for absolute abundances $\{n_i(t)\}$ the four phenotypes:

$$\begin{aligned}
\frac{\partial n_P}{\partial t} &= -S(t) n_P(t), \\
\frac{\partial n_F}{\partial t} &= S(t)\sigma^{(P\to F)} n_P(t), \\
\frac{\partial n_D}{\partial t} &= X^{(D)}(1-\varphi^{(D\to H)}) n_D(t) + S(t)\sigma^{(P\to D)} n_P(t), \\
\frac{\partial n_H}{\partial t} &= X^{(H)} n_H(t) + X^{(D)}\varphi^{(D\to H)} n_D(t) + S(t)\sigma^{(P\to H)} n_P(t).
\end{aligned} \tag{F.2}$$

We now will show that these equations admit an exact solution in terms of special functions. Since the healthy cells depend on healthy, damaged, and persister cells; the damaged cells depend on damaged and persister cells; the failed cells depend on the failed cells; and the persister cells only depend on persister cells, it will make most sense to solve the equations in order for $P \to F, D \to H$.

First, we note the persister cell dynamics are solvable from separation of variables, as done by Fang and Allison [36]:

$$\begin{aligned}
\int_{n_P(0)}^{n_P(t)} \frac{\mathrm{d}n}{n} &= -\int_0^t \mathrm{d}t'\, \alpha e^{\beta t'} \\
\Rightarrow \quad \log \frac{n_P(t)}{n_P(0)} &= -c\left(e^{\beta t} - 1\right),
\end{aligned} \tag{F.3}$$

bioRxiv preprint doi: https://doi.org/10.64898/2026.03.15.711953; this version posted March 16, 2026. The copyright holder for this preprint (which was not certified by peer review) is the author/funder, who has granted bioRxiv a license to display the preprint in perpetuity. It is made available under aCC-BY 4.0 International license.

where we have defined $c \equiv \alpha/\beta$ for future notational convenience. Rearranging, we have

$$n_P(t) = n_P(0)e^{-c\left(e^{\beta t}-1\right)}. \tag{F.4}$$

Now, substituting the first line of eq. (F.2), into the second line, we find

$$\frac{\partial n_F}{\partial t} = -\sigma^{(P\to F)}\frac{\partial n_P}{\partial t}, \tag{F.5}$$

which we then integrate:

$$\begin{aligned} \int_0^t \mathrm{d}t'\,\frac{\partial n_F}{\partial t'} &= -\sigma^{(P\to F)}\int_0^t \mathrm{d}t'\,\frac{\partial n_P}{\partial t'} \\ \Rightarrow\quad n_F(t) - n_F(0) &= -\sigma^{(P\to F)}\left[n_P(t) - n_P(0)\right]. \end{aligned} \tag{F.6}$$

Rerranging, this gives us

$$n_F(t) = n_F(0) + \sigma^{(P\to F)}\left[n_P(0) - n_P(t)\right]. \tag{F.7}$$

Next, we define $a = X^{(D)}\left(1-\varphi^{(D\to H)}\right)$ for convenience and rearrange the third line of eq. (F.2) to obtain

$$\begin{aligned} \frac{\partial n_D}{\partial t} - a n_D(t) &= S(t)\sigma^{(P\to D)}n_P(t) \\ &= \alpha e^{\beta t}\sigma^{(P\to D)}n_P(0)e^{-c\left(e^{\beta t}-1\right)}, \end{aligned} \tag{F.8}$$

where in the second line we have substituted in the previously solved result for $n_P(t)$. Now, we note that

$$\begin{aligned} \frac{\partial}{\partial t}\left(e^{-at}n_D(t)\right) &= e^{-at}\frac{\partial n_D}{\partial t} - e^{-at}a n_D(t) \\ &= e^{-at}\left(\frac{\partial n_D}{\partial t} - a n_D(t)\right) \\ &= e^{-at}\left(\alpha e^{\beta t}\sigma^{(P\to D)}n_P(0)e^{-c\left(e^{\beta t}-1\right)}\right) \\ &= \alpha\sigma^{(P\to D)}n_P(0)e^{(\beta-a)t-c\left(e^{\beta t}-1\right)}. \end{aligned} \tag{F.9}$$

Integrating both sides, we have

$$\begin{aligned} \int_0^t \mathrm{d}t'\,\frac{\partial}{\partial t'}\left(e^{-at'}n_D(t')\right) &= \alpha\sigma^{(P\to D)}n_P(0)\int_0^t \mathrm{d}t'\,e^{(\beta-a)t'-c\left(e^{\beta t'}-1\right)} \\ \Rightarrow\quad e^{-at}n_D(t) - n_D(0) &= \alpha\sigma^{(P\to D)}n_P(0)\int_0^t \mathrm{d}t'\,e^{(\beta-a)t'-c\left(e^{\beta t'}-1\right)} \\ \Rightarrow\quad n_D(t) &= e^{at}\left[n_D(0) + \alpha\sigma^{(P\to D)}n_P(0)\int_0^t \mathrm{d}t'\,e^{(\beta-a)t'-c\left(e^{\beta t'}-1\right)}\right]. \end{aligned} \tag{F.10}$$

Now, we evaluate the integral. First, we perform the u substitution by setting $u = ce^{\beta t'}$, which gives us $\mathrm{d}u = \beta u\,\mathrm{d}t'$ or $\mathrm{d}t' = \mathrm{d}u/(\beta u)$. We also have $e^{\beta u} = u/c$ and $t' = \beta^{-1}\log(u/c)$. It then follows that

$$\begin{aligned} e^{(\beta-a)t'-c\left(e^{\beta t'}-1\right)}\,\mathrm{d}t' &= e^{(\beta-a)t'-c\left(\frac{u}{c}-1\right)}\frac{\mathrm{d}u}{\beta u} \\ &= \frac{e^c}{\beta}c^{\frac{a}{\beta}-1}u^{-a/\beta}e^{-u}\,\mathrm{d}u. \end{aligned} \tag{F.11}$$

Now, we can rewrite the integral

$$\int_0^t \mathrm{d}t'\,e^{(\beta-a)t'-c\left(e^{\beta t'}-1\right)} = \frac{e^c}{\beta}c^{\frac{a}{\beta}-1}\int_c^{ce^{\beta}t}\mathrm{d}u\,u^{-a/\beta}e^{-u}. \tag{F.12}$$

If we let $q_D = 1-\alpha/\beta$, we can rewrite the integral as

$$\int_c^{ce^{\beta}t}\mathrm{d}u\,u^{-a/\beta}e^{-u} = \int_c^{ce^{\beta}t}\mathrm{d}u\,u^{q_D-1}e^{-u}. \tag{F.13}$$

bioRxiv preprint doi: https://doi.org/10.64898/2026.03.15.711953; this version posted March 16, 2026. The copyright holder for this preprint (which was not certified by peer review) is the author/funder, who has granted bioRxiv a license to display the preprint in perpetuity. It is made available under aCC-BY 4.0 International license.

Using the definition of the lower incomplete gamma function

$$\gamma(s,x) = \int_0^x \mathrm{d}v\, v^{s-1} e^{-v}, \tag{F.14}$$

we can now write

$$\int_c^{ce^{\beta t}} \mathrm{d}u\, u^{q_D-1} e^{-u} = \gamma\left(q_D, ce^{\beta t}\right) - \gamma\left(q_d, c\right). \tag{F.15}$$

Thus, we have

$$n_D(t) = e^{at}\left\{n_D(0) + \alpha\sigma^{(P\to D)} n_P(0)\beta^{-1} e^c c^{-q_D}\left[\gamma\left(q_D, ce^{\beta t}\right) - \gamma\left(q_D, c\right)\right]\right\}. \tag{F.16}$$

Finally, we turn to the dynamics of the healthy cells. We define a linear combination of the healthy and damaged cells

$$y(t) \equiv n_H(t) + k n_D(t), \tag{F.17}$$

which, upon differentiation, gives

$$\frac{\partial y}{\partial t} = \frac{\partial n_H}{\partial t} + k\frac{\partial n_D}{\partial t}. \tag{F.18}$$

Substituting in the equations from eq. (F.2), this becomes

$$\frac{\partial y}{\partial t} = X^{(H)} n_H(t) + X^{(D)}\varphi^{(D\to H)} n_D(t) + S(t)\sigma^{(P\to H)} n_P(t) + k\left[a n_D(t) + S(t)\sigma^{(P\to D)} n_P(t)\right]. \tag{F.19}$$

Substituting back in $n_H(t) = y(t) - k n_D(t)$ and simplifying, we have

$$\frac{\partial y}{\partial t} = X^{(H)} y(t) + \left[-X^{(H)}k + X^{(D)}\varphi^{(D\to)} + ka\right] n_D(t) + S(t)\left(\sigma^{(P\to H)} + k\sigma^{(P\to D)}\right) n_P(t). \tag{F.20}$$

If we can make the bracket vanish, then $\frac{\partial y}{\partial t}$ only depends on terms which can be written again in terms of the lower incomplete gamma function. So, we try to find the k which makes the bracket vanish

$$-X^{(H)}k + X^{(D)}\varphi^{(D\to)} + ka = 0, \tag{F.21}$$

which yields

$$k = \frac{X^{(D)}\varphi^{(D\to)}}{X^{(H)} - a}. \tag{F.22}$$

Defining $B \equiv \sigma^{(P\to H)} + k\sigma^{(P\to D)}$ for convenience, we can now write

$$\begin{aligned} \frac{\partial y}{\partial t} &= X^{(H)} y(t) + BS(t) n_P(t) = X^{(H)} y(t) + B\alpha e^{\beta t} n_P(t) \\ \Rightarrow] \quad \frac{\partial y}{\partial t} &- X^{(H)} y(t) = B\alpha e^{\beta t} n_P(t) \end{aligned} \tag{F.23}$$

Now, noting that

$$\begin{aligned} \frac{\partial}{\partial t}\left(e^{-X^{(H)}t} y(t)\right) &= e^{-X^{(H)}t}\frac{\partial y}{\partial t} - e^{-X^{(H)}t} X^{(H)} y(t) \\ &= e^{-X^{(H)}t}\left(\frac{\partial y}{\partial t} - X^{(H)} y(t)\right) \\ &= e^{-X^{(H)}t}\left(B\alpha e^{\beta t} n_P(t)\right). \end{aligned} \tag{F.24}$$

Integrating both sides, we have

$$\begin{aligned} \int_0^t \mathrm{d}t'\, \frac{\partial}{\partial t'}\left(e^{-X^{(H)}t'} y(t')\right) &= \int_0^t \mathrm{d}t'\, e^{-X^{(H)}t'}\left(B\alpha e^{\beta t'} n_P(t')\right) \\ \Rightarrow \quad e^{-X^{(H)}t} y(t) - y(0) &= \alpha B n_P(0)\int_0^t \mathrm{d}t'\, e^{\left(\beta - X^{(H)}\right)t' - c\left(e^{\beta t'} - 1\right)}. \end{aligned} \tag{F.25}$$

We can solve the integral using the same u substitution as before, except now we have $X^{(H)}$ in place of a. So, defining $q_H \equiv 1 - X^{(H)}\beta$, we can write the integral as

$$\int_0^t \mathrm{d}t'\, e^{\left(\beta - X^{(H)}\right)t' - c\left(e^{\beta t'} - 1\right)} = \frac{e^c}{\beta} c^{-q_H}\left[\gamma\left(q_H, ce^{\beta t}\right) - \gamma\left(q_H, c\right)\right]. \tag{F.26}$$

bioRxiv preprint doi: https://doi.org/10.64898/2026.03.15.711953; this version posted March 16, 2026. The copyright holder for this preprint (which was not certified by peer review) is the author/funder, who has granted bioRxiv a license to display the preprint in perpetuity. It is made available under aCC-BY 4.0 International license.

Finally, we have

$$y(t) = e^{X^{(H)}t} \left\{ y(0) + \alpha B n_P(0) \beta^{-1} e^c c^{-q_H} \left[\gamma \left(q_H, ce^{\beta t} \right) - \gamma \left(q_H, c \right) \right] \right\}, \tag{F.27}$$

with $y(0) = n_H(0) + kn_D(0)$. Now, replacing $y(t)$ using the solutions to $n_D(t)$ and eq. (F.17), we have the abundance of healthy cells

$$\begin{aligned} n_H(t) = {} & e^{X^{(H)}t} \left\{ n_H(0) + kn_D(0) + \alpha B n_P(0) \beta^{-1} e^c c^{-q_H} \left[\gamma \left(q_H, ce^{\beta t} \right) - \gamma \left(q_H, c \right) \right] \right\} \\ & - ke^{at} \left\{ n_D(0) + \alpha \sigma^{(P \to D)} n_P(0) \beta^{-1} e^c c^{-q_D} \left[\gamma \left(q_D, ce^{\beta t} \right) - \gamma \left(q_D, c \right) \right] \right\}. \end{aligned} \tag{F.28}$$
